# Comparative CRISPRi screens reveal a human stem cell dependence on mRNA translation-coupled quality control

**DOI:** 10.1038/s41594-025-01616-3

**Published:** 2025-07-11

**Authors:** Geraldine Rodschinka, Sergio Forcelloni, Felix M. Kühner, Sascha Wani, Henrick Riemenschneider, Dieter Edbauer, Andrew Behrens, Danny D. Nedialkova

**Affiliations:** 1 Mechanisms of Protein Biogenesis Laboratory, Max Planck Institute of Biochemistry, Martinsried, Germany; 2 German Center for Neurodegenerative Diseases (DZNE), Munich, Germany; 3 Munich Cluster of Systems Neurology (SyNergy), Munich, Germany; 4 Department of Bioscience, TUM School of Natural Sciences, Technical University of Munich, Garching, Germany

**Keywords:** Translation, Pluripotent stem cells, Systems biology

## Abstract

The translation of mRNA into proteins in multicellular organisms needs to be carefully tuned to changing proteome demands in development and differentiation, while defects in translation often have a disproportionate impact in distinct cell types. Here we used inducible CRISPR interference screens to compare the essentiality of genes with functions in mRNA translation in human induced pluripotent stem cells (hiPS cells) and hiPS cell-derived neural and cardiac cells. We find that core components of the mRNA translation machinery are broadly essential but the consequences of perturbing translation-coupled quality control factors are cell type dependent. Human stem cells critically depend on pathways that detect and rescue slow or stalled ribosomes and on the E3 ligase ZNF598 to resolve a distinct type of ribosome collision at translation start sites on endogenous mRNAs with highly efficient initiation. Our findings underscore the importance of cell identity for deciphering the molecular mechanisms of translational control in metazoans.

## Abstract (web summary)

By probing the essentiality of diverse genes encoding mRNA translation factors in different human cell types using CRISPR interference screens, Rodschinka et al. discover that human stem cells critically depend on proteins rescuing stalled or collided ribosomes.

## Main

The human genome contains ~20,000 predicted protein-coding genes but only half are expressed at a time^1^ and fewer than one fifth are expressed at similar levels in all cell types^2^. In metazoans, intricately orchestrated developmental programs generate cell types with highly specialized functions and distinct protein content. This poses unique challenges to the mRNA translation machinery, which needs to accommodate rapid shifts in global and specific protein demands during developmental transitions^3^ and to faithfully synthesize even extremely long proteins such as titin (~34,000 aa) in millions of copies in specialized cell types^4^. As the length of human proteins varies over 400-fold and their intracellular abundance spans six orders of magnitude^5^, quality control pathways can mitigate errors in mRNA translation and dispose of problematic mRNAs and incomplete nascent chains that could be toxic to cells^6,7^. Failure of these surveillance mechanisms has been linked to human neurological disorders^8^.

The complexity of the mRNA translation machinery makes it challenging to determine how its plasticity is achieved during development, a task that is further compounded by the early embryonic lethality upon constitutive knockout of its components in mammals. Studies using conditional knockouts have shown that perturbations in mRNA translation can have differential effects in distinct cell types^9–11^ but the underlying mechanisms are debated^12,13^. The physiological signals that activate translation quality control pathways remain equally enigmatic and their mechanistic dissection has so far relied on reporters mimicking problematic mRNAs^6,7^.

The function of human genes has been probed in high throughput in a range of transformed and cancer-derived cell lines^14^ but these models are of limited value for dissecting cell-type-specific regulation because of their genetic heterogeneity and aberrant gene expression^15,16^. Translational control is also extensively rewired to sustain the rapid proliferation of cancer cells^17^. Human induced pluripotent stem cells (hiPS cells), by contrast, can self-renew without transformation and can give rise to nearly any cell type in culture^18^. Recent advances enable the robust differentiation of hiPS cells along distinct cell lineages and CRISPR–Cas9-based functional genomics in these models^19–21^. Here we took advantage of these technologies to define the essentiality of 262 genes encoding core and regulatory mRNA translation machinery components with CRISPR interference (CRISPRi) screens. In contrast to the nearly universal essentiality of core ribosomal proteins (r-proteins) and translation factors, the depletion of many proteins that mediate translation-coupled quality control was detrimental only in some cell contexts. Such divergent genetic dependencies were especially pronounced in pathways that detect and rescue slow or stalled ribosomes. We identified distinct stress responses upon perturbing ribosome rescue pathways in different cellular contexts and discovered a novel role for the ribosome quality control sensor ZNF598 (refs. ^22–25^) in detecting ribosome collisions during translation initiation on endogenous mRNAs in human stem cells. Our study establishes an experimental platform for dissecting the cell-context-dependent determinants of fundamental biological processes.

## Comparative screens in human cells by inducible CRISPRi

**Fig. 1:**
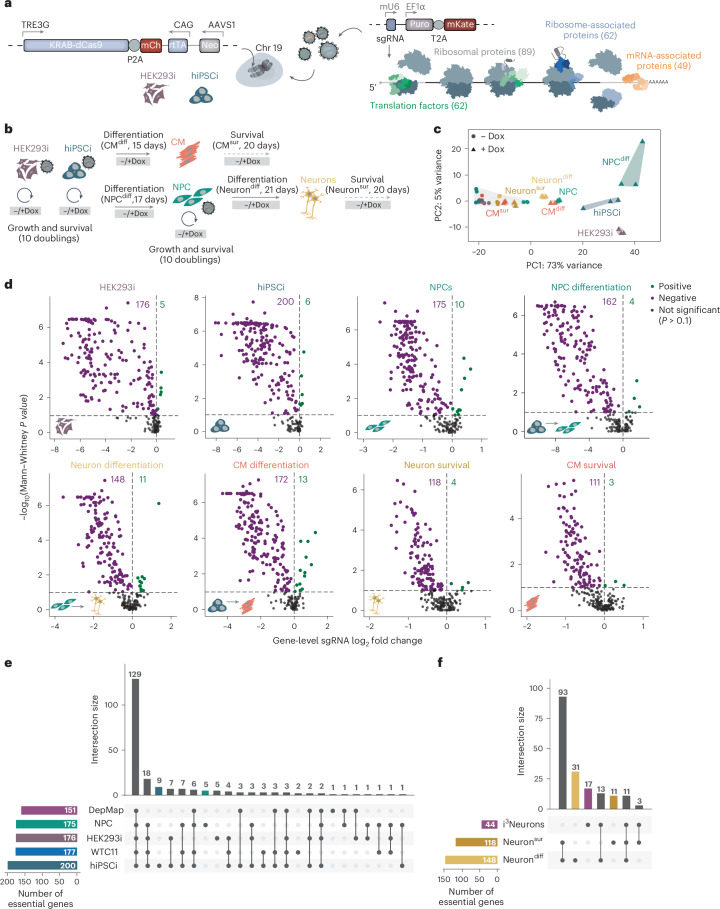
Comparative inducible CRISPRi screens identify essential components of the mRNA translation machinery in human cells. **a**, Schematic of inducible CRISPRi cell line generation and screening. Right, gene counts for major functional groups of the human translation machinery included in the sgRNA library. Neo, neomycin resistance gene; mCh, mCherry; Puro, puromycin resistance gene; KRAB, Krüppel associated box; rtTA, reverse tetracycline-controlled transactivator; mKate, monomeric Kate2 protein. **b**, Schematic of the workflow of inducible hiPS cell differentiation into NPCs, neurons and CMs and the different types and durations of inducible CRISPRi screens. **c**, Principal component analysis of variance stabilizing-transformed count data for sgRNAs from DESeq2 (*n* = 2 biological replicates for CM screens; *n* = 3 biological replicates for inducible hiPS cell, inducible HEK293 cell, NPC and neuron screens). PC, principal component; diff, differentiation; sur, survival. **d**, Volcano plots of gene-level sgRNA log_2_ fold change (mean of the top three sgRNAs per gene by magnitude) and *P* values (from comparisons of all sgRNAs targeting a given gene to all negative control sgRNAs) for each screening condition relative to matched uninduced (−Dox) controls. Genes with significant (two-sided Mann–Whitney test, *P* ≤ 0.1) positive or negative enrichment scores are shown in green and purple, respectively. **e**,**f**, UpSet plots showing overlap of genes with significant (two-sided Mann–Whitney test, *P* ≤ 0.1) negative enrichment scores in inducible hiPS cell, inducible HEK293 cell and NPC screens in comparison to common essential genes in cancer cell lines (DepMap^14^) and genome-wide CRISPRi in the WTC11 hiPS cell line^35^ (**e**) and neuron differentiation or survival screens compared to a genome-wide CRISPRi screen in WTC11-derived i^3^Neurons^36^ (**f**). hiPSCi, inducible hiPS cell; HEK293i, inducible HEK293 cell. Source data

**Extended Data Fig. 1:**
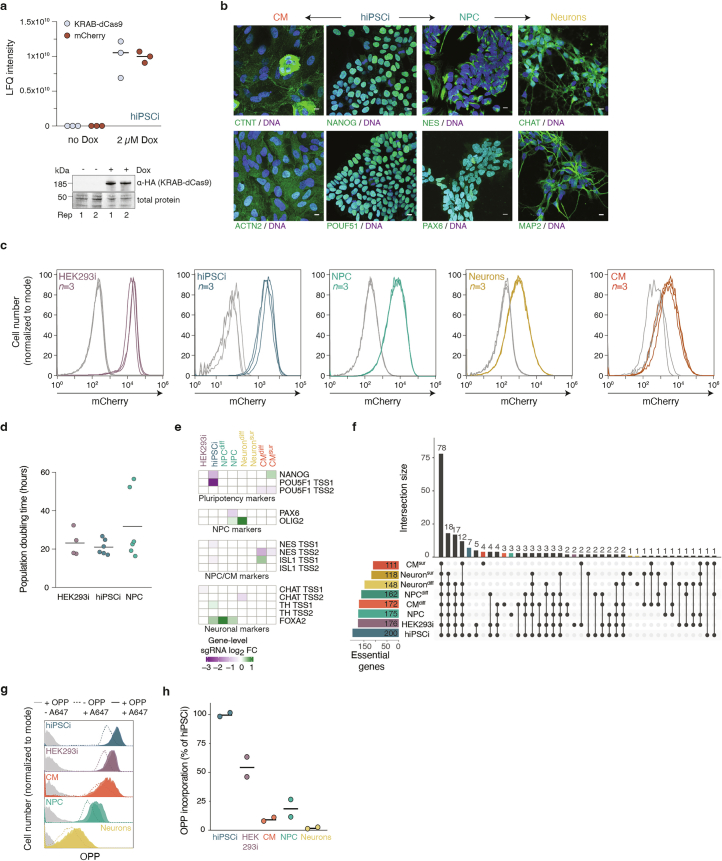
Workflow for comparative genetic screens by inducible CRISPRi. **a**, LFQ intensities of KRAB-dCas9 and mCherry protein levels in hiPSCi +/- doxycycline (Dox) measured by mass spectrometry (*n* = 3 biological replicates, top) and immunoblot analysis of HA-tagged KRAB-dCas9 in control hiPSCi and after treatment with 2 µM doxycycline for 2 days (bottom). **b**, Immunostaining for cell type-specific markers (green) and DAPI (blue). Scale bar: 10 µm. **c**, Quantification of % mCherry-positive cells by flow cytometry (*n* = 3 biological replicates) after KRAB-dCas9 induction with 2 µM doxycycline for two days (in hiPSCi and HEK293i), 5 passages (NPC), and for five days in differentiated neurons and CM. **d**, Quantification of cell doubling time (*n* = 2 biological replicates for HEK293i; *n* = 3 biological replicates for hiPSCi and NPC). **e**, Heatmaps of significant gene-level sgRNA log_2_ fold change (FC) (two-sided Mann–Whitney test *P* ≤ 0.1) for cell identity markers. diff = differentiation, sur = survival, TSS = transcriptional start site. **f**, UpSet plot of overlap among genes with significant negative gene-level sgRNA log_2_ FC (two-sided MannWhitney test *P* ≤ 0.1). **g,h**, Global protein synthesis measurements by O-propargyl-puromycin (OPP) labeling. (**g**) Median fluorescence intensity quantified by flow cytometry ( > 10,000 cells/ analysis); (**h**) Data from (**e**) normalized to a matched control not treated with OPP (-OPP + A647) and calculated as a fraction of the average signal in hiPSCi (*n* = 2 biological replicates for +OPP + A647; *n* = 1 biological replicate for -OPP + A647 and +OPP-A647). Source data

We profiled the reliance of different human cell types on genes encoding components of the translational machinery by inducible CRISPRi screening^26,27^. Because it does not introduce double-stranded DNA (dsDNA) breaks, CRISPRi does not trigger p53-mediated toxicity, which is an obstacle to genetic screening in human pluripotent stem cells^28^. We used a previously validated workflow^27^ that relies on the insertion of a doxycycline-inducible KRAB–dCas9 expression cassette at the *AAVS1* safe harbor locus in the reference *kucg-2* hiPS cell line^29,30^ and in HEK293 for comparison (hereafter referred to as inducible hiPS cells and inducible HEK293 cells). We confirmed that KRAB–dCas9 was undetectable in inducible hiPS cells cultured without doxycycline (Extended Data Fig. 1a). We used CRISPRiaDesign^31^ to design a pool of single guide RNAs (sgRNAs) targeting the promoters of 262 human genes encoding core and regulatory mRNA translation factors, as well as nine cell-specific marker genes as controls. The resulting library (3,000 sequences, including 10% nontargeting controls) was cloned in a lentiviral expression vector (Fig. 1a).

We used our established protocols to differentiate inducible hiPS cells into neural progenitor cells (NPCs), neurons and cardiomyocytes (CMs)^30^ (Fig. 1b), which comprise cell types and developmental transitions subject to extensive translational control^3,32–34^. Consistent with our previous data from parental *kucg-2* hiPS cells^30^, lineage-specific markers were uniformly expressed in inducible hiPS cells (*NANOG* and *POU5F1*) and their differentiated counterparts (*PAX6* and *NES* in NPCs, *CHAT* and *MAP2* in neurons and *CTNT* and *ACTN2* in CMs) (Extended Data Fig. 1b). Analysis of mCherry levels as a proxy for KRAB–dCas9 expression (Fig. 1a) revealed a robust induction upon doxycycline addition to inducible HEK293 cells, inducible hiPS cells, NPCs, neurons and CMs (Extended Data Fig. 1c**)**.

To compare gene essentiality across cellular contexts, we transduced inducible hiPS cells, NPCs and inducible HEK293 cells with the lentiviral sgRNA library while ensuring that only one sgRNA is expressed in each cell and collected matched samples grown without or with doxycycline after ten population doubling times (Extended Data Fig. 1d). We also assessed gene essentiality during or after the derivation of NPCs and CMs from transduced hiPS cells and neurons from transduced NPCs (Fig. 1b). Principal component analysis of sgRNA counts revealed a clustering of uninduced controls, as well as biological replicates of individual screens (Fig. 1c). By calculating gene-level enrichment or depletion scores with an established CRISPRi screen analysis pipeline^31^, we found between 111 and 200 targets that were significantly depleted (Mann–Whitney *P* ≤ 0.1) and 3–13 targets significantly enriched in the different screens (Fig. 1d). Notably, sgRNAs targeting cell markers were depleted only in the relevant screens (Extended Data Fig. 1e).

Of the 262 genes we targeted, 151 were identified as ‘common essential’ in over 500 human cancer cell lines (DepMap)^14^. Accordingly, 150 of these 151 genes (99%) were also negative hits in our screens in dividing cells (inducible HEK293 cells, inducible hiPS cells and NPCs; Fig. 1e). Furthermore, 177 of the 262 targeted genes scored as negative hits in a genome-wide CRISPRi screen in the WTC11 hiPS cell line^35^ and we observed concordant negative phenotypes in inducible hiPS cells for 175 of those 177 (99%; Fig. 1e). We found that 27 genes (15%) were essential in our *kucg-2*-derived inducible hiPS cells but not in WTC11 hiPS cells, which may reflect differences in genetic background or screen setup. Additionally, 148 genes were essential during neuronal differentiation and 118 genes were essential for neuron survival (Fig. 1f). By contrast, only 44 of our 262 targets previously scored as essential in a genome-wide screen in WTC11-derived i^3^Neurons^36^ and we recovered 24 of them (55%). Our inducible CRISPRi screens, thus, recapitulate known genetic dependencies and identify novel ones in hiPS cells and their derived cell types.

A comparison across all screening contexts revealed that inducible hiPS cells had the highest sensitivity to mRNA translation perturbations, with 200 of 262 (76%) genes scoring as essential compared to 176 and 175 (67%) in inducible HEK293 cells and NPC, respectively (Extended Data Fig. 1f). This could in part be linked to the exceptionally high global protein synthesis rates in inducible hiPS cells (Extended Data Fig. 1g,h). The lower overall number of hits in the neuron and CM survival screens may, at least in part, be because of less efficient protein depletion in nondividing cells because of a lack of dilution by cell division^37^. Genetic dependencies specific for a single cell type were extremely rare; only one gene essential for the survival of neurons (*NAA11*) or CM survival (*CPEB2*) and four genes essential for inducible HEK293 cell growth (*CARHSP1*, *EIF4E3*, *EIF4G3* and *IGF2BP2*) did not score as hits in any other of our screens (Supplementary Table 1).

**Extended Data Fig. 2:**
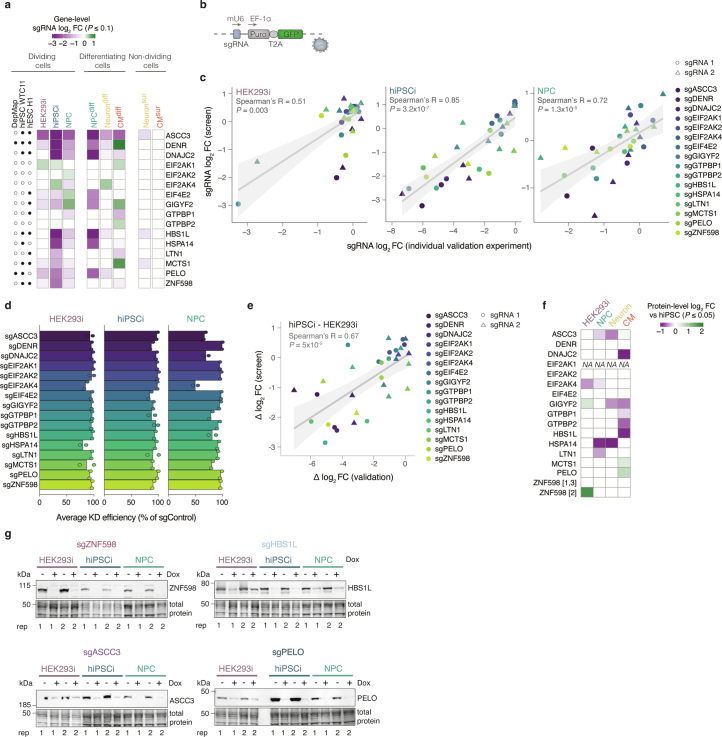
Validation of pooled CRISPRi screens. **a**, Heatmaps of gene-level sgRNA log_2_ FC for 16 genes selected for validation; white: not significant (two-sided Mann–Whitney test *P* > 0.1). **b**, Schematic of individual sgRNA expression construct. Puro: puromycin resistance gene. **c**, Correlation of single sgRNA phenotype from growth competition assays [log_2_ (-/+Dox)] and log_2_ FC of the same sgRNA in the pooled CRISPRi screen (Spearman’s R, *P* < 0.05) for the two most active sgRNAs (sgRNA1 and sgRNA2) for each gene in the screens. Solid lines denote linear regression model; grey shading denotes 95% confidence interval. **d**, Knockdown efficiency of genes from (**a**) compared to a non-targeting control (sgControl) measured by quantitative RT-PCR for the most active sgRNA from the screens (sgRNA1; *n* = 2 biological replicates, each with *n* = 3 technical replicates). **e**, Correlation plot of cell type specificity (hiPSCi log_2_ FC minus HEK293i log_2_ FC) calculated for individual sgRNAs from growth competition assays [∆log_2_ FC (validation), *n* = 2 biological replicates] and the same sgRNAs in the pooled CRISPRi screen [∆log_2_ FC (screen); *n* = 3 biological replicates]. Solid line denotes linear regression model; grey shading denotes 95% confidence interval. **f**, Heatmaps of significant (pairwise two-tailed t-test, FDR < 0.01) protein-level log_2_ FC for screening targets in (**a**) measured by mass spectrometry in the indicated cell types in comparison to hiPSCi (*n* = 3 biological replicates; numbers in brackets indicate isoforms). **g**, Immunoblot analysis of ZNF598, ASCC3, HBS1L, and PELO in sgRNA-transduced cells after KRAB-dCas9 induction ( + Dox) and in matched uninduced controls (-Dox). Source data

To validate the screen results, we selected the two most highly effective sgRNAs targeting 16 genes with differential essentiality in dividing cells (Extended Data Fig. 2a) and transduced them individually in inducible hiPS cells, NPCs and inducible HEK293 cells (Extended Data Fig. 2b). sgRNA enrichment or depletion in these individual experiments was highly correlated with the scores for the same sgRNAs in the screens (Spearman’s *R* = 0.85 for inducible hiPS cells, 0.72 for NPCs and 0.51 for inducible HEK293 cells; Extended Data Fig. 2c) and reverse transcription (RT)–qPCR confirmed efficient knockdown for all 16 targets in all three cell lines (Extended Data Fig. 2d). As a measure of hiPS cell versus HEK293 phenotype specificity, we calculated a cell type specificity score for each of these 16 genes. We found a significant positive correlation between the cell type specificity scores computed from the screen and from the single-guide validation experiments (Spearman’s *R* = 0.67; Extended Data Fig. 2e). The proteins encoded by these 16 genes were present at similar levels in inducible hiPS cells, NPCs and inducible HEK393 cells according to quantitative mass spectrometry (MS). An exception to this was ZNF598, which was ~2-fold more abundant in inducible HEK293 cells (Extended Data Fig. 2f), possibly because of aneuploidy of this cell line^16^.

As human stem cells have higher proteasome activity than somatic cells^38^, we considered that other cell types may appear more resilient to gene repression because of inefficient protein depletion. However, immunoblot analysis of four target gene products (ZNF598, ASCC3, HBS1L and PELO) revealed that they were all depleted to a similar extent in inducible hiPS cells, NPCs and inducible HEK293 cells (Extended Data Fig. 2g). These data suggest that the differential sensitivity of these cell types to the same genetic perturbation reflects distinct demands on the protein synthesis machinery.

## Broad essentiality of the core translation machinery

**Fig. 2:**
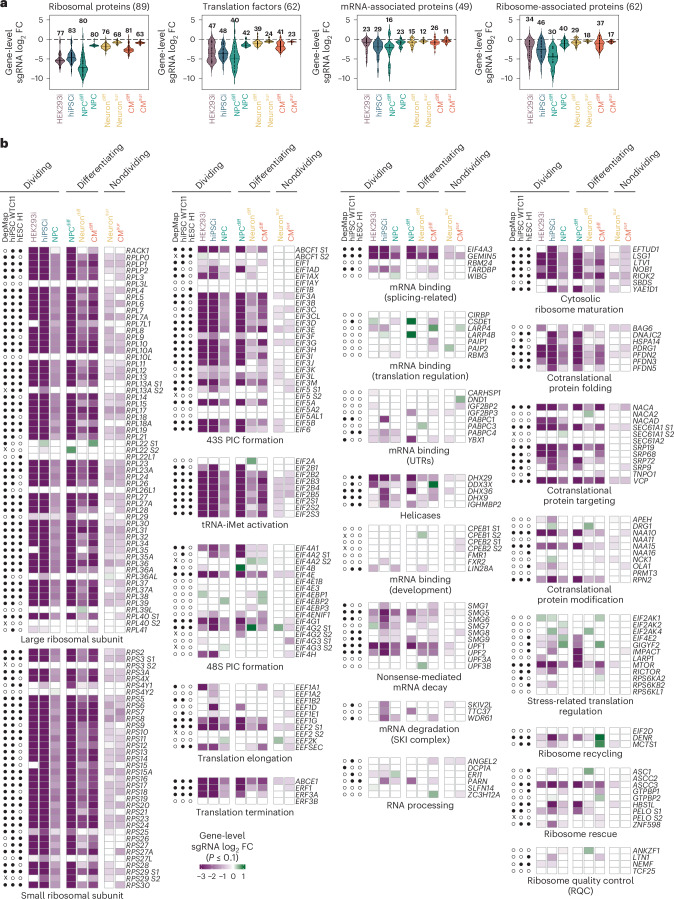
Common and cell-type-specific effects of mRNA translation perturbations in human cells. **a**, Violin plots of gene-level sgRNA log_2_ fold changes (mean of the top three sgRNAs per gene by magnitude) in each screen separated into functional gene groups (r-proteins, translation factors, mRNA-associated proteins and ribosome-associated proteins). The number of genes with significant (two-sided Mann–Whitney test, *P* ≤ 0.1) enrichment or depletion in each screen is indicated above each violin. FC, fold change. **b**, Heatmaps of data in **a**. Significant (two-sided Mann–Whitney test, *P* ≤ 0.1) gene-level enrichment or depletion in each screen indicated by color. White, not significant in the respective screen. Data for genes with more than one annotated TSS (S1 and S2) were analyzed separately. Open and closed circles indicate nonessential and essential genes, respectively, in the common essential gene set from DepMap 23Q4 (ref. ^14^) and genome-wide CRISPRi screens in WTC11 hiPS cells and H1 hES cells^35^; x indicates the absence of data for alternative TSSs in DepMap. hESC, human embryonic stem cells; PIC, preinitiation complex; tRNA-iMet, initiator tRNA methionine; SKI, superkiller. Source data

**Extended Data Fig. 3:**
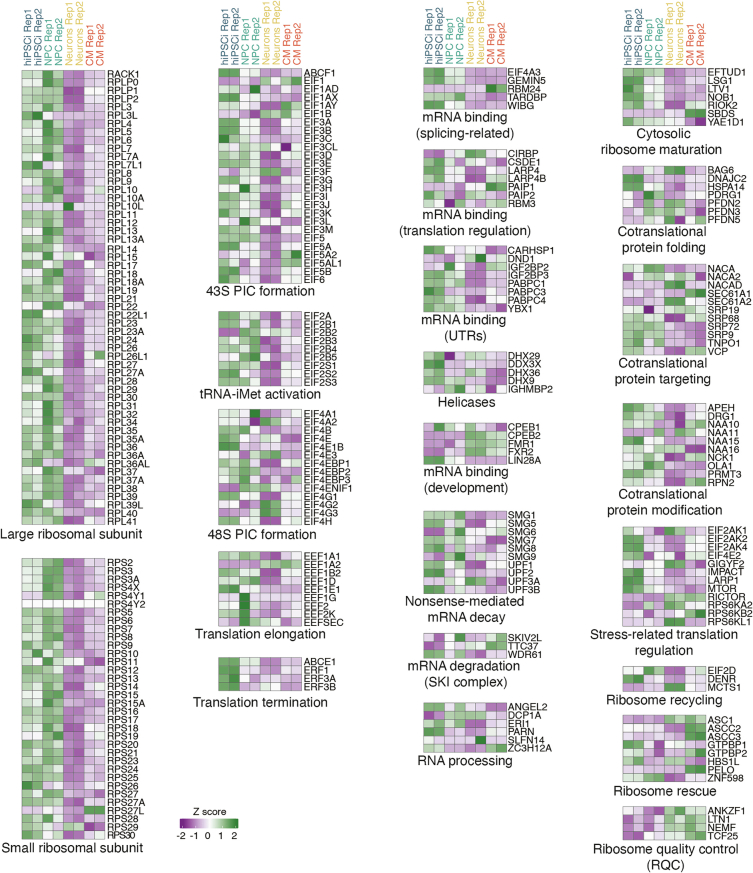
mRNA expression levels for screen targets in hiPSC and hiPSC-derived cells. Gene expression heatmaps for screen targets in parental *kucg-2* hiPSC and NPC, neurons and CM cultures differentiated from them (*n* = 2 biological replicates). Standardized Z scores were calculated from DESeq2-normalized RNA-Seq gene counts across samples (data from ref. ^30^). Source data

To determine how perturbations in distinct functional modules of mRNA translation impact different cell types, we broadly grouped gene targets into r-proteins, translation factors (initiation, elongation or termination), mRNA-associated proteins and ribosome-associated proteins. We found strong negative effects of depleting nearly all canonical r-proteins and translation factors in dividing cells (inducible hiPS cells, NPCs and inducible HEK293 cells) and during differentiation (Fig. 2a). Furthermore, 76 of the 79 canonical r-protein genes were essential in dividing cells and 63 were essential across all cell contexts (Fig. 2a,b). These data were consistent with the core essentiality of these genes in DepMap^14^ and in genome-wide CRISPRi screens in WTC11 hiPS cells and the H1 human embryonic stem cell (hES cell) line^35^ (Fig. 2b). Depletion of ribosome export and maturation factors (LSG1, LTV1 and RIOK2) also had concordant strongly negative effects in all screens (Fig. 2b). Together, these data indicate that, despite the global downregulation of ribosome biogenesis and core translation-related genes that accompanies differentiation (Extended Data Fig. 3)^39^, new ribosomes must be continuously produced in nondividing cell types.

Two of the small ribosomal subunit (40S) and seven of the large ribosomal subunit (60S) r-protein-encoding genes have paralogs, some of which may function in specific tissues^40,41^. Knockdown of *RPL22* or its paralog *RPL22L1* had minimal effects in all screens (Fig. 2b), consistent with their functional redundancy and the compensatory expression of *Rpl22l1* in *Rpl22*^*−/−*^ mice^42^. *RPS27L* and *RPL36AL* scored as essential in a subset of our screens (Fig. 2b), suggesting that their protein products function in selected cell types.

We also found divergent effects upon knockdown of gene paralogs for initiation and elongation factors. Knockdown of *EEF1A2*, the brain-specific and muscle-specific paralog of the ubiquitously expressed elongation factor-encoding *EEF1A1* (ref. ^43^), inhibited neuron survival (Fig. 2b). Repression of the initiation factor eIF4G1 paralog *EIF4G2* inhibited growth and survival in dividing cells (HEK293 cells, hiPS cells and NPCs) but surprisingly promoted neuron survival (Fig. 2b). The depletion of proteins that globally repress translation initiation downstream of growth or stress signaling (eIF4EBPs or EIF2AKs) similarly promoted cell proliferation or survival in specific contexts (Fig. 2b). Collectively, these findings demonstrate the ability of our comparative screens to identify common and cell-context-specific genetic dependencies.

## Stem cell sensitivity to translation quality control defects

In contrast to the broad essentiality of genes encoding the core translation machinery, we found surprisingly divergent genetic dependencies for many genes encoding translation quality control factors. hiPS cells were particularly sensitive to the depletion of factors mediating the rescue of ribosomes that stall or collide when translating problematic mRNA or peptide sequences^9^ (Fig. 2b). For example, depletion of PELO and its partner HBS1L, which destabilize ribosomes stalled at the 3′ end of truncated mRNA reporters^44–48^, strongly inhibited inducible hiPS cell growth and neural differentiation. While inducible HEK293 cells were also moderately sensitive to PELO loss, they were surprisingly resilient to HBS1L depletion (Fig. 2b), supporting prior data that the two proteins may function independently in some cellular contexts^10,49^.

**Extended Data Fig. 4:**
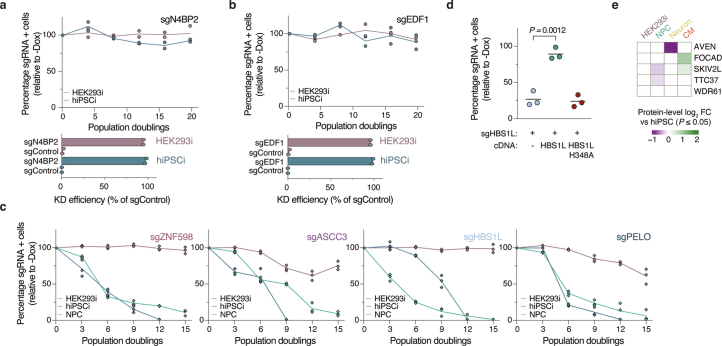
Validation experiments with individual sgRNAs. **a,b**, Growth assays of HEK293i or hiPSCi expressing a sgRNA targeting N4BP2 (**a**) or EDF1 (**b**) (*n* = 2 biological replicates; line: average) and knockdown efficiency measurements of target genes compared to a non-targeting control (sgControl) by quantitative RT-PCR (*n* = 2 biological replicates, each with *n* = 3 technical replicates). **c**, Growth assays of cells transduced with the second most potent sgRNA (sgRNA2) targeting *ZNF598*, *ASCC3*, *HBS1L*, or *PELO* in the hiPSCi screen (*n* = 3 biological replicates; line: average). The percentage of GFP-positive (GFP + ) cells was measured by flow cytometry ( > 10,000 cells/ analysis) every three population doublings and normalized to GFP+ cell numbers in matched uninduced (-Dox) controls. **d**, Growth assays of hiPSCi transduced with a sgRNA targeting *HBS1L* alone or in combination with a cDNA encoding wild-type or a GTPase-inactive *HBS1L* (H348A) (*n* = 3 biological replicates, *P*-values from unpaired two-tailed t-test). **e**, Heatmaps of significant (pairwise two-tailed t-test, FDR < 0.01) protein-level log_2_ FC for SKI complex components in the indicated cell types in comparison to hiPSCi measured by mass spectrometry (*n* = 3 biological replicates). Source data

Unresolved ribosome stalling can lead to ribosome collisions, which trigger distinct quality control and stress response pathways^50^. Collided ribosomes can be recognized by the E3 ligase ZNF598, which ubiquitinates the 40S r-proteins uS10 and eS10 (refs. ^22,23,25,51–53^) to enable ribosome disassembly by the helicase ASCC3 (refs. ^54,55^). While *ASCC3* was near-universally essential in our screens, *ZNF598* knockdown was highly detrimental for inducible hiPS cells and, to a lesser extent, NPC growth, while it was better tolerated by inducible HEK293 cells (Fig. 2b). Notably, *ZNF598* also scored as essential in genome-wide CRISPRi screens in WTC11 hiPS cells and H1 hES cells^35^ (Fig. 2b). We note that not all factors implicated in ribosome rescue were essential in inducible hiPS cells; depletion of N4BP2, the homolog of yeast Cue2 and worm NONU-1 that cleave mRNAs in the A site of collided ribosomes^56,57^, or EDF1, which binds collided ribosomes^58,59^, had no appreciable effect on either inducible hiPS cell or inducible HEK293 cell growth and survival (Extended Data Fig. 4a,b). These data indicate that the functional importance of translation-coupled quality control strongly depends on the cellular context and is disproportionally high in human stem cells.

**Fig. 3:**
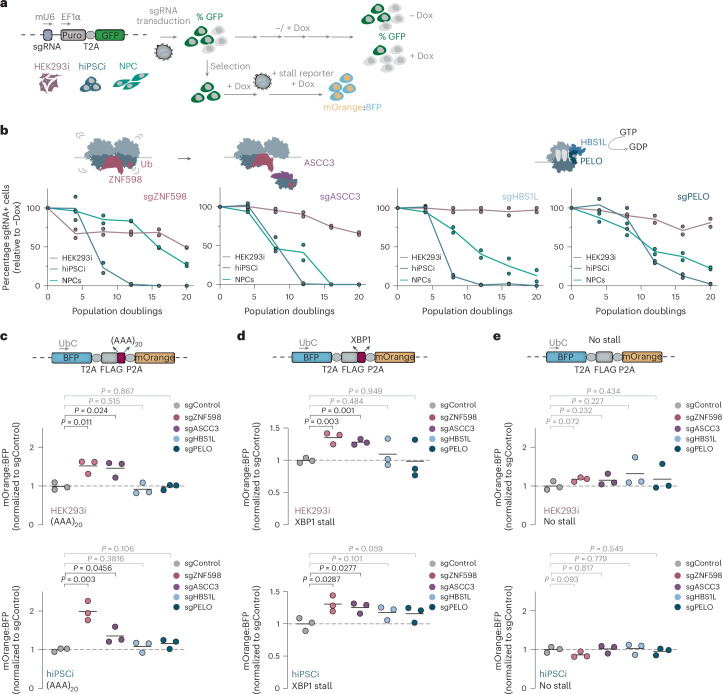
Differential resilience to perturbed ribosome rescue is not because of functional redundancy. **a**, Schematic of expression constructs and workflows for growth competition and stalling reporter readthrough assays. **b**, Growth assays of cells transduced with the most potent sgRNA targeting *ZNF598*, *ASCC3*, *HBS1L* or *PELO* in the inducible hiPS cell screen (*n* = 2 biological replicates; line, mean). The percentage of GFP-positive (GFP^+^) cells was measured by flow cytometry (>10,000 cells per analysis) every four population doublings and normalized to GFP^+^ cell numbers in matched uninduced (−Dox) controls. GTP, guanosine triphosphate; GDP, guanosine diphosphate. **c**–**e**, Stalling readthrough of reporters containing (AAA)_20_ (**c**), the XBP1 arrest peptide (**d**) and a no-stall control (**e**). The median fluorescence intensity for BFP and mOrange was quantified by flow cytometry (>20,000 cells per analysis) in the indicated cell lines transduced with the most potent sgRNA targeting *ZNF598*, *ASCC3*, *HBS1L* or *PELO* based on the inducible hiPS cell screen. The ratio of mOrange to BFP in knockdown cells was normalized to that for the same reporter in cells transduced with a nontargeting sgRNA (sgControl; *n* = 3 biological replicates; *P* values from an unpaired two-tailed *t*-test). UbC, ubiquitin C promoter; FLAG, Flag-tag. Source data

To test whether the resilience of some cells to ribosome rescue factor depletion is linked to potential functional redundancies in specific cell contexts, we further characterized the impact of knocking down *ZNF598*, *ASCC3*, *PELO* or *HBS1L*. In individual sgRNA experiments, the knockdown of these four genes was highly detrimental for inducible hiPS cell growth and survival, while it was much better tolerated by inducible HEK293 cells (Fig. 3a,b and Extended Data Fig. 4c). Because the *HBS1L* locus also encodes SKI7, a cytoplasmic exosome cofactor expressed from an alternative isoform^60,61^, we asked whether the essential role of *HBS1L* in inducible hiPS cells could be partially attributed to SKI7 depletion. However, reintroducing complementary DNA (cDNA) encoding wild-type *HBS1L* but not a GTPase-deficient HBS1L mutant rescued the growth defects of *HBS1L* knockdown in inducible hiPS cells (Extended Data Fig. 4d), suggesting that the GTPase activity of HBS1L is essential for human stem cell proliferation and survival. Cancer cells with genetic defects that compromise the SKI complex were recently shown to rely on the PELO–HBS1L complex for survival^62,63^. However, we observed similar levels of core SKI complex proteins and their stabilizing partners FOCAD and AVEN in inducible hiPS cells^29,30^ and inducible HEK293 cells (Extended Data Fig. 4e), indicating that HBS1L depletion does not impair stem cell physiology because of SKI complex deficiency.

Because the endogenous translation events targeted by ZNF598, ASCC3, PELO or HBS1L are poorly defined, we measured readthrough of model ribosome stalling substrates upon knockdown of each of these genes with an established fluorescence-based quantitative reporter assay^22,24^. We analyzed the ratio of mOrange to blue fluorescent protein (BFP) produced from reporters containing one of two well-characterized ribosome stalling sequences: an AAA-encoded stretch of 20 lysines ((AAA)_20_), which mimics ribosome encounters with prematurely polyadenylated mRNA^22,24^, or the XBP1 arrest peptide^54,64–66^. In line with previous reports^24,55^, ZNF598 or ASCC3 depletion increased the readthrough of (AAA)_20_ and XBP1 stalls in both inducible hiPS cells and inducible HEK293 cells (Fig. 3c–e). These data suggest that the higher resilience of inducible HEK293 cells to the loss of ZNF598 or ASCC3 in comparison to inducible hiPS cells is not because of functional redundancies linked to rescuing ribosomes stalled on problematic sequences within open reading frames (ORFs).

## Ribosome rescue averts a cytotoxic ISR in human stem cells

**Fig. 4:**
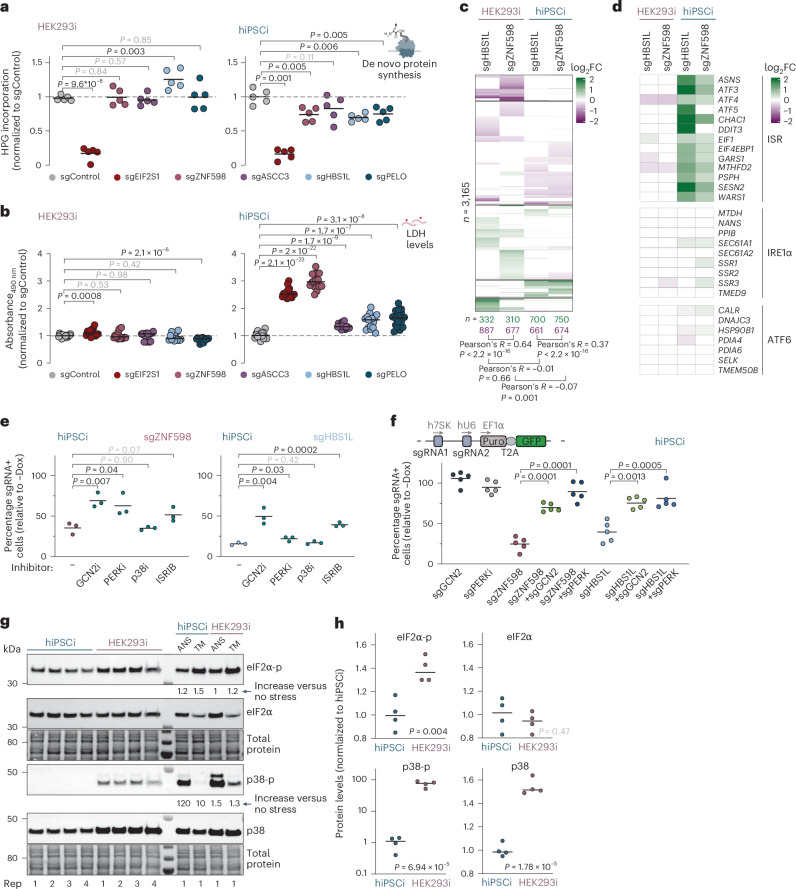
Ribosome rescue prevents a cytotoxic ISR in human stem cells. **a**, Global protein synthesis measurements in knockdown inducible hiPS cells or inducible HEK293 cells by HPG labeling for 30 min. Median fluorescence intensity was quantified by flow cytometry (>10,000 cells per analysis) and normalized to values from cells transduced with sgControl (*n* = 5 biological replicates; *P* values from an unpaired two-tailed *t*-test). **b**, LDH measurements in culture supernatants from knockdown inducible hiPS cells or inducible HEK293 cells. Values were normalized to supernatant from cells transduced with sgControl (*n* = 4 technical replicates and 4 biological replicates; *P* values from an unpaired two-sided *t*-test). **c**, Heatmap of mRNAs differentially expressed upon *ZNF598* or *HBS1L* repression in inducible hiPS cells or inducible HEK293 cells (*n* = 2 biological replicates; two-sided Wald test with Benjamini–Hochberg correction, adjusted *P* ≤ 0.05; *n* = 3165). **d**, Heatmap of a data subset from **c** showing genes upregulated downstream of ATF4 within the ISR or downstream of ATF6 or IRE1α upon ER protein folding perturbations^70^. **e**, Growth assays of inducible hiPS cells expressing an sgRNA targeting *ZNF598* (day 6) or *HBS1L* (day 8) in the absence (−) or presence of GCN2i (A-92, 1.25 µM), PERKi (GSK2606414, 4 nM), p38i (SB203580, 1 µM) or ISRIB (50 nM) in comparison to uninduced controls (*n* = 3 biological replicates; *P* values from an unpaired two-tailed *t*-test). **f**, Growth assays of inducible hiPS cells expressing an sgRNA targeting *ZNF598*, *HBS1L*, *GCN2* and *PERK* alone or in combination in comparison to uninduced controls (*n* = 5 biological replicates; *P* values from an unpaired two-tailed *t*-test). **g**, Immunoblot analysis of eIF2α phosphorylation, p38 phosphorylation and total eIF2α and p38 levels in inducible hiPS cells and inducible HEK293 cells (*n* = 4 biological replicates). Inducible hiPS cells treated with 2.5 µM tunicamycin (TM) for 2 h served as a positive control for induction of eIF2α phosphorylation; inducible hiPS cells treated with 0.05 mg L^−1^ ANS for 15 min served as a positive control for induction of p38 phosphorylation. Rep, replicate. **h**, Quantification of signal intensity in **g** by densitometry (*P* values from an unpaired two-tailed *t*-test). Source data

**Extended Data Fig. 5:**
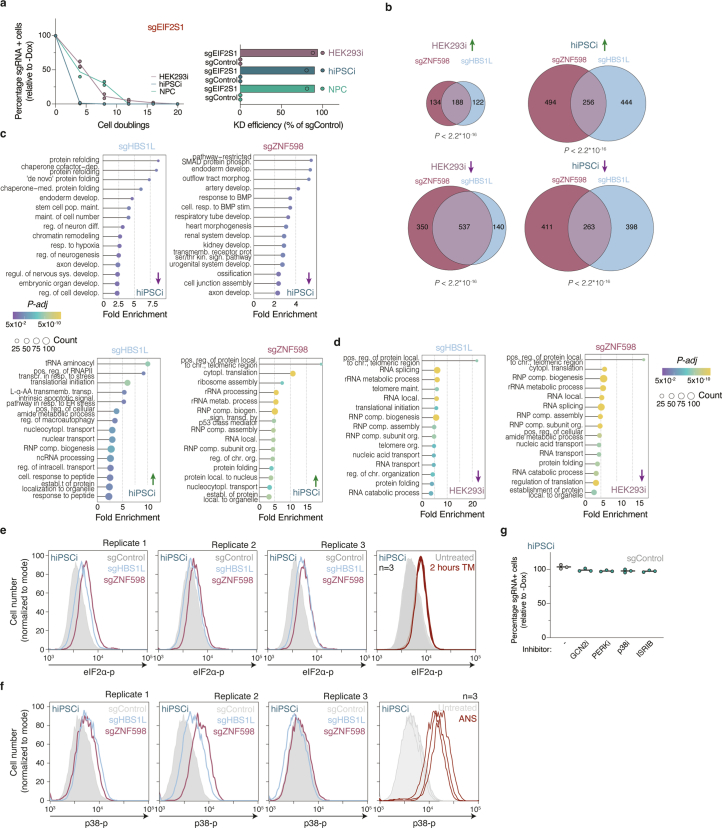
Analysis of stress pathway activation in different cellular contexts. **a**, Growth assays of cells transduced with a sgRNA targeting *EIF2S1* in HEK293i, hiPSCi, or NPC cells (*n* = 2 biological replicates; line: average) and knockdown efficiency measurements of target genes compared to a non-targeting control (sgControl) by quantitative RT-PCR (*n* = 2 biological replicates, each with *n* = 3 technical replicates). **b**, Venn diagrams of overlap between genes upregulated or downregulated in HEK293i or hiPSCi depleted for ZNF598 or HBS1L by CRISPRi (*p*-values from one-tailed Fisher’s exact test). **c,d**, Gene ontology (GO) enrichment in differentially expressed mRNAs using the “Biological Process” function in ClusterProfiler (one-tailed Fisher’s exact test with Benjamini-Hochberg correction, *P-adj* ≤ 0.01) in knockdown hiPSCi (**b**) or HEK293i (**c**). **e, f**, Flow cytometry analysis of eIF2α phosphorylation (**d**) and p38 phosphorylation (**e**) upon depletion of ZNF598 or HBS1L in hiPSCi in comparison to cells transduced with a non-targeting sgRNA (sgControl; n = 3 biological replicates; matched samples). hiPSC treated with 2.5 µg tunicamycin (TM) for 2 hours to induce the ISR or with 0.05 mg/l anisomycin (ANS) for 15 minutes to induce the RSR served as a positive control. **g**, Growth assays of hiPSCi expressing a non-targeting sgRNA (sgControl, day 6) in the absence (-) or presence of inhibitors of GCN2 (GCN2i, A-92, 1.25 µM), PERK (PERKi, GSK2606414, 4 nM), p38 (p38i, SB203580, 1 µM), or the ISR (ISRIB, 50 nM) in comparison to uninduced controls (*n* = 3 biological replicates). Source data

To define the mechanisms underlying the human stem cell-specific growth defects upon ribosome rescue perturbations, we next examined how these perturbations impact global translation rates and cell viability. Depleting the core essential initiation factor EIF2S1 (Extended Data Fig. 5a) reduced de novo protein synthesis in both inducible HEK293 cells and inducible hiPS cells by >80%. Strikingly, depletion of ZNF598, ASCC3, HBS1L or PELO significantly reduced de novo protein synthesis in inducible hiPS cells but not in inducible HEK293 cells (Fig. 4a). Lactate dehydrogenase (LDH) levels in culture medium, a proxy for cytotoxicity^67^, were also significantly elevated upon knockdown of ribosome rescue factors in hiPS cells but not in inducible HEK293 cells (Fig. 4b). These data indicate that perturbed ribosome rescue induces cytotoxicity selectively in the stem cell context.

We next profiled the gene expression changes induced by knockdown of *HBS1L* or *ZNF598* in inducible hiPS cells and inducible HEK293 cells by RNA sequencing (RNA-seq). The patterns of gene expression changes were strikingly similar among the two genetic perturbations but differed substantially between the two cell lines (Fig. 4c, Extended Data Fig. 5b and Supplementary Table 2). Genes upregulated in inducible hiPS cells were enriched for Gene Ontology (GO) terms related to chromosome organization, RNA processing and transport and protein folding and localization (Extended Data Fig. 5c), whereas inducible HEK293 cells exhibited downregulation of many genes with the same GO terms (Extended Data Fig. 5d). Notably, knockdown of either *ZNF598* or *HBS1L* resulted in a marked upregulation of integrated stress response (ISR) marker genes in inducible hiPS cells but not in inducible HEK293 cells (Fig. 4d). In mammals, the ISR is activated by one of four kinases (GCN2, PERK, PKR or HRI), which sense a broad and partially overlapping range of stresses and phosphorylate the α subunit of eIF2 (ref. ^68^). This leads to a global repression of cap-dependent mRNA translation but selectively increases *ATF4* translation. ATF4, in turn, induces a specific gene expression program to restore cellular homeostasis or trigger cell death^68,69^. ATF4 target genes^69,70^ were significantly upregulated upon depletion of ZNF598 or HBS1L in inducible hiPS cells but not in inducible HEK293 cells, while the expression of other response genes downstream of other stress sensors (for example, for protein misfolding in the endoplasmic reticulum (ER): ATF6 and IRE1α)^70^ was largely unchanged (Fig. 4d). In line with the ATF4-dependent gene expression signatures, *ZNF598* or *HBS1L* knockdown increased eIF2α phosphorylation levels in inducible hiPS cells (Extended Data Fig. 5e). Apart from inducing the ISR, unresolved ribosome stalling and collisions can trigger the ribotoxic stress response (RSR) through phosphorylation of the MAP kinase p38 by ZAKα (refs. ^50,71^). The levels of phosphorylated p38 were increased in inducible hiPS cells depleted of ZNF598 or HBS1L, albeit to a variable extent (Extended Data Fig. 5f), indicating that the loss of ribosome rescue factors in human stem cells results in a modest activation of the RSR.

To test whether the impaired cell growth (Fig. 3b) and cytotoxicity (Fig. 4b) of *HBS1L* or *ZNF598* knockdown in inducible hiPS cells can be attributed to the ISR or RSR, we transduced cells with sgRNAs targeting each of these genes and induced KRAB–dCas9 expression in the presence of chemical inhibitors of GCN2, PERK and p38 (GCN2i, PERKi and p38i). We also treated cells with integrated stress response inhibitor (ISRIB), which dampens the ISR downstream of eIF2α phosphorylation^72^ at intermediate activation levels^68,73^. GCN2i, PERKi and ISRIB alleviated the growth and survival defects of *ZNF598* or *HBS1L* knockdown in inducible hiPS cells (Fig. 4e) without affecting cells transduced with a nontargeting sgRNA (Extended Data Fig. 5g). Codepleting GCN2 or PERK using dual sgRNA vectors also alleviated the growth and survival defects imparted by ZNF598 or HBS1L depletion in inducible hiPS cells **(**Fig. 4f). Collectively, these data indicate that the loss of ribosome rescue factors in human stem cells impairs cell growth and survival primarily through the ISR.

We next sought to evaluate the molecular basis for the lack of global translation defects (Fig. 4a), cytotoxicity (Fig. 4b) and ISR gene expression signatures (Fig. 4d) upon ZNF598 or HBS1L depletion in inducible HEK293 cells. Unlike inducible hiPS cells, inducible HEK293 cells are aneuploid^16^ and may, thus, experience constitutive proteotoxic stress because of unbalanced gene expression^74^, making them more resilient to additional perturbations^75^. Strikingly, the levels of phosphorylated eIF2α were ~40% higher in inducible HEK293 cells, an effect size comparable to the eIF2α phosphorylation increase triggered by pharmacological induction of ER stress with tunicamycin (Fig. 4g,h). These data are in line with the lower de novo protein synthesis rates we observed in inducible HEK293 cells compared to inducible hiPS cells (Extended Data Fig. 1g,h). Moreover, the levels of phosphorylated p38 were ~100-fold higher in inducible HEK293 cells than in inducible hiPS cells (Fig. 4g,h). Taken together, these data suggest that constitutive stress signaling may mask the phenotypic consequences of perturbing ribosome rescue in aneuploid cell lines like HEK293.

## ZNF598 defects cause start site ribosome pauses in stem cells

**Fig. 5:**
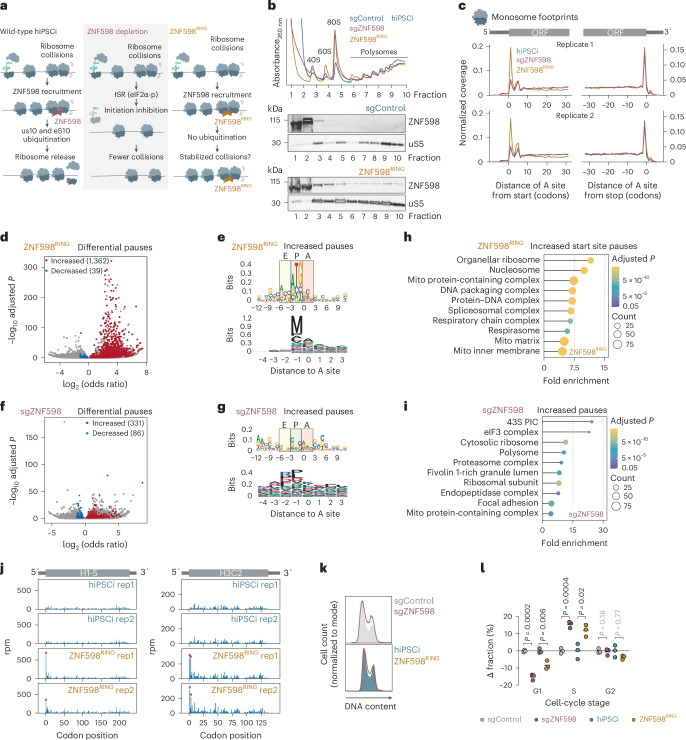
Defective ubiquitination by ZNF598 in human stem cells elicits ribosome pausing at start sites. **a**, Schematic model of the consequences of ZNF598 depletion or ZNF598^RING^ expression in inducible hiPS cells. **b**, Polysome profiles (top) and immunoblot analysis of ZNF598 and uS5 in polysome gradient fractions (bottom) of inducible hiPS cells expressing sgControl, a *ZNF598* sgRNA or ZNF598^RING^ (*n* = 1 biological replicate). **c**, Metagene profiles of ribosomal A-site occupancy from monosome footprints around CDS start and stop sites (*n* = 2 biological replicates). **d**, Volcano plot of differential ribosome pause sites upon ZNF598^RING^ expression in inducible hiPS cells (two-tailed Fisher’s exact test with Benjamini–Hochberg correction, adjusted *P* ≤ 0.01). **e**, Nucleotide (top) and amino acid (bottom) motif analysis of significantly increased pause sites in well-translated mRNAs (>0.5 footprints per codon in all samples; *n* = 3,421) in ZNF598^RING^-expressing inducible hiPS cells. **f**, Volcano plot of differential ribosome pausing analysis upon *ZNF598* knockdown (sgZNF598) in inducible hiPS cells as in **d**. **g**, Nucleotide (top) and amino acid (bottom) motif analysis of significantly increased pause sites in well-translated mRNAs (>0.5 footprints per codon in all samples; *n* = 2,463) in sgZNF598 inducible hiPS cells. **h**,**i**, GO term enrichment analysis of genes with significantly increased pause sites (one-tailed Fisher’s exact test with Benjamini–Hochberg correction, adjusted *P* ≤ 0.01) within the first five codons in ZNF598^RING^-expressing inducible hiPS cells (**h**) and throughout the ORF in sgZNF598 inducible hiPS cells (**i**) filtered for TPM > 1 in RNA-seq from inducible hiPS cells. **j**, Distribution of monosome footprints (in reads per million (rpm)) along the H1-5 (left) and H3C2 (right) mRNA in control and ZNF598^RING^-expressing inducible hiPS cells (*n* = 2 biological replicates). Significant differential pauses are indicated with red arrows. **k**, Representative histograms of cell-cycle analysis in inducible hiPS cells by DNA staining with EdU followed by flow cytometry. **l**, Changes in the fraction of cells in different cell-cycle phases calculated by flow cytometry analysis after EdU staining (*n* = 3 biological replicates; >10,000 cells per analysis; *P* values from an unpaired two-tailed *t*-test). Source data

The pronounced sensitivity of human stem cells to ZNF598 depletion suggests that some endogenous mRNAs are difficult to translate and are, thus, subject to ZNF598-dependent quality control in this cellular context. These events may be challenging to detect in wild-type cells because ZNF598-mediated r-protein ubiquitylation likely occurs on a millisecond scale^76^ and is followed by rapid ribosome disassembly^53^. They may also be challenging to detect upon ZNF598 depletion because the knockdown of ribosome rescue factors commonly activated the ISR in human stem cells (Fig. 4c,d)^77–79^. The resulting global inhibition of translation initiation because of eIF2α phosphorylation (Extended Data Fig. 5e) may mask the ribosome stalling or collision events on endogenous mRNAs that need to be rescued by each pathway (Fig. 5a). Indeed, deletion of *HEL2*, the yeast homolog of *ZNF598*, also triggers the ISR and concomitantly decreases ribosome collision frequency^80,81^.

**Extended Data Fig. 6:**
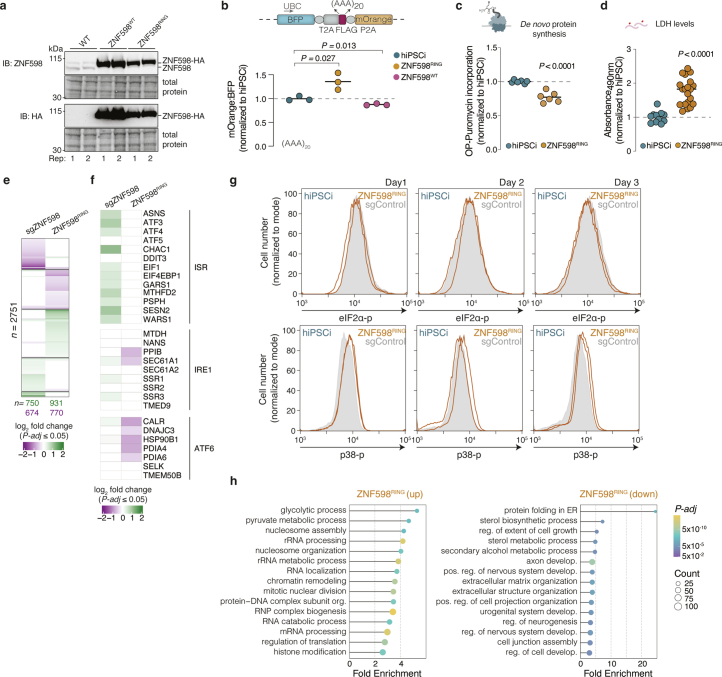
ZNF598^RING^ expression in human stem cells induces cytotoxicity but not through the ISR. **a**, Immunoblot analysis of wild-type hiPSCi and hiPSC transduced with cDNA encoding HA-tagged wild-type (ZNF598^WT^) or C29S/C32S (ZNF598^RING^) *ZNF598* (*n* = 2 biological replicates). **b**, Stalling readthrough of reporters containing an AAA-encoded stretch of twenty lysines (AAA_20_) in control hiPSCi and hiPSCi transduced with ZNF598^WT^ or ZNF598^RING^ cDNA. The median fluorescence intensity for BFP and mOrange was quantified by flow cytometry ( > 20,000 cells/ analysis) and normalized to average values for the hiPSCi control (*n* = 3 biological replicates; *P*-values from unpaired two-tailed t-test). **c**, Global protein synthesis measurements by OP-Puromycin and AF647 detection. Median fluorescence intensity was quantified by flow cytometry ( > 10,000 cells/ analysis) and normalized to average values for the control (*n* = 6 biological replicates; *p*-values from unpaired two-tailed t-test). **d**, Lactate dehydrogenase (LDH) measurements in culture supernatants from wild type or ZNF598^RING^-expressing hiPSCi two days after transduction. Values were normalized to supernatant from wild type hiPSCi (*n* = 4 technical replicates and 5 biological replicates; *P*-values from unpaired two-tailed t-test). **e,f**, Differential gene expression analysis in hiPSCi upon ZNF598 knockdown (sgZNF598) in comparison to a non-targeting sgRNA (sgControl), or in ZNF598^RING^-expressing hiPSCi in comparison to untransduced hiPSCi (*n* = 2 biological replicates). (**e**) Heatmap of mRNAs differentially expressed in at least one context (two-sided Wald test; *P-adj* ≤ 0.05; *n* = 2289). (**f**) Heatmap of a subset from (**e**) showing genes associated with the ISR. **g**, Flow cytometry analysis of eIF2α (top) or p38 (bottom) phosphorylation upon ZNF598^RING^ expression in hiPSCi in comparison to cells transduced with a non-targeting sgRNA (sgControl; *n* = 2 biological replicates) on day 1-3 after transduction. **h**, Gene ontology (GO) enrichment in differentially expressed mRNAs from (**e**) using the “Biological Process” function in ClusterProfiler (one-tailed Fisher’s exact test with Benjamini-Hochberg correction, *P-adj* ≤ 0.01). Source data

We postulated that a catalytically inactive ZNF598 would stabilize ribosomes engaged in problematic translation events on endogenous mRNAs. To test this, we substituted the conserved cysteines in the RING domain of ZNF598, which ablate uS10 and eS10 ubiquitination without impairing substrate recognition and act in a dominant-negative manner in HEK293T cells^22,23,54^. Overexpression of a C29S;C32S RING domain mutant (ZNF598^RING^) but not wild-type ZNF598 increased the expression of mOrange downstream of the (AAA)_20_ stalling sequence in inducible hiPS cells (Extended Data Fig. 6a,b), confirming a dominant-negative role for ZNF598^RING^ in human stem cells. ZNF598^RING^ cosedimented robustly with inducible hiPS cell polysomes (Fig. 5b), in line with prior observations of a more stable association of ZNF598 and Hel2 RING mutants with polysomes in HEK293T cells and yeast, respectively^22,82^. These data suggest that ZNF598 dissociates less efficiently from its substrates in the absence of r-protein ubiquitination.

Similarly to *ZNF598* knockdown, ZNF598^RING^ expression in inducible hiPS cells decreased de novo protein synthesis rates (Fig. 5b and Extended Data Fig. 6c) and resulted in significantly elevated LDH levels (Extended Data Fig. 6d**)**, indicating that defective ZNF598 ubiquitination is cytotoxic in this cellular context. The gene expression changes induced by ZNF598^RING^ in inducible hiPS cells, however, were distinct from those triggered by ZNF598 depletion. Although the levels of more than 1,500 mRNAs were significantly altered, ATF4 target genes were not among them (Extended Data Fig. 6e,f and Supplementary Table 3). eIF2α phosphorylation levels were also not appreciably elevated in ZNF598^RING^-expressing inducible hiPS cells, while p38 phosphorylation was only mildly increased (Extended Data Fig. 6g). Genes upregulated in ZNF598^RING^-expressing inducible hiPS cells were enriched for GO terms related to metabolism, nucleosome assembly and organization and RNA processing, while downregulated genes were related to cell growth and development (Extended Data Fig. 6h). ZNF598^RING^ expression, therefore, does not activate the ISR in human stem cells.

**Extended Data Fig. 7:**
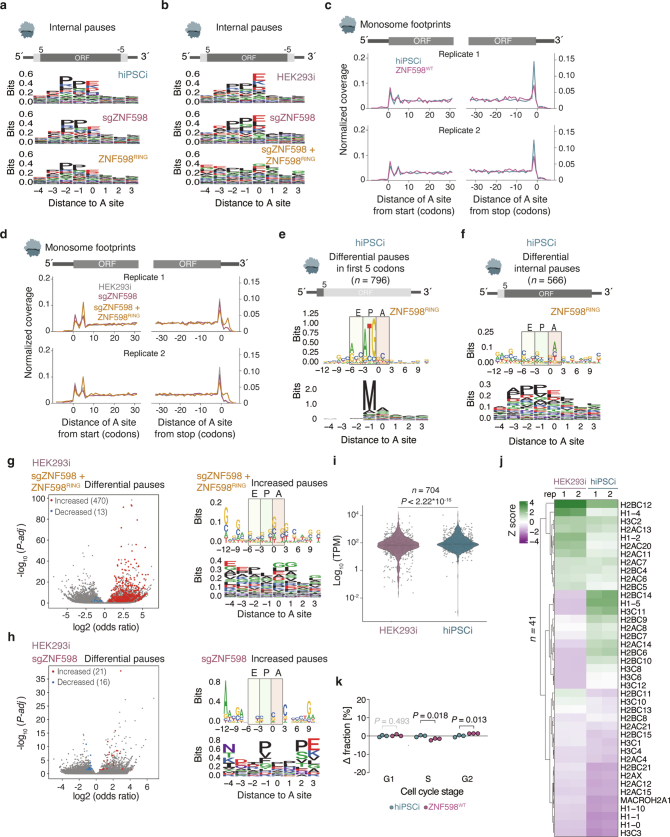
ZNF598^RING^ increases ribosome occupancy at translation start sites in human stem cells. **a,b**, Amino acid motif analysis of internal pause sites (excluding the first and last five codons of each CDS) in wild type, *ZNF598* knockdown (sgZNF598), and ZNF598^RING^-expressing hiPSCi (**a**) or HEK293i (**b**). **c**, Metagene profiles of ribosomal A sites from monosome footprints around CDS start and stop codons in wild type and ZNF598^WT^-overexpressing hiPSCi (*n* = 2 biological replicates). **d**, Metagene profiles of ribosomal A sites from monosome footprints around CDS start and stop codons in wild type, sgZNF598 and ZNF598^RING^-expressing HEK293i (*n* = 2 biological replicates). **e,f**, Nucleotide (top) and amino acid (bottom) motif analysis of significantly increased pause sites within (**e**) or excluding (**f**) the first five codons in ZNF598^RING^-expressing hiPSCi (two-tailed Fisher’s exact test with Benjamini-Hochberg correction, *P-adj* ≤ 0.05). **g**, Volcano plot of differential ribosome pause sites upon ZNF598^RING^ expression in HEK293i (two-tailed Fisher’s exact test with Benjamini-Hochberg correction, *P-adj* ≤ 0.05) (left). Nucleotide (top) and amino acid (bottom) motif analysis of significantly increased pause sites in well-translated mRNAs ( > 0.5 footprints/codon in all samples, *n* = 1438) in ZNF598^RING^-expressing HEK293i (right). **h**, Volcano plot of differential ribosome pausing analysis upon *ZNF598* knockdown (sgZNF598) in HEK293i as in (**g**, left). Nucleotide (top) and amino acid (bottom) motif analysis of significantly increased pause sites in well-translated mRNAs ( > 0.5 footprints/codon in all samples, *n* = 1003) in sgZNF598 HEK293 as in (**g**, right). **i**, Violin plots (center line: median) of TPM values from RNA-Seq for genes with significantly increased A-site pausing within the first 5 codons in ZNF598^RING^-expressing hiPSCi (*n* = 704) in sgControl HEK293i and hiPSCi (*P*-value from two-sided Wilcoxon test). **j**, Hierarchically clustered heatmap of scaled *Z* scores of normalized unique transcript counts (TPM) from RNA-Seq in sgControl HEK293i and hiPSCi (*n* = 2 biological replicates) for histone mRNAs with increased A-site pausing within the first 5 codons in ZNF598^RING^-expressing hiPSCi (*n* = 41). **k**, Changes in the fraction of cells in different cell cycle phases calculated by flow cytometry analysis after EdU staining (*n* = 3 biological replicates, >10,000 cells/ analysis; *P*-values from unpaired two-tailed t-test). Source data

To determine how ZNF598 perturbations impact translation, we analyzed ribosome occupancy by high-throughput sequencing of 20–32-nt ribosome-protected mRNA footprints from inducible hiPS cells and inducible HEK293 cells depleted for ZNF598 or expressing ZNF598^RING^. Because the basal levels of ZNF598 were ~2-fold higher in inducible HEK293 cells (Extended Data Fig. 2f,g), we expressed ZNF598^RING^ in cells depleted for endogenous ZNF598 by CRISPRi. Motif analysis of internal ribosomal pauses (*Z* score ≥ 5)^83^ in these monosome footprints revealed the typical enrichment of proline-rich stretches across all conditions^84^ (Extended Data Fig. 7a,b), confirming our ability to capture known instances of slow elongation.

We found a striking increase in monosome footprint density close to annotated coding sequence (CDS) start sites upon ZNF598^RING^ overexpression in hiPS cells. This was not detectable upon ZNF598 depletion (Fig. 5c), wild-type ZNF598 overexpression (Extended Data Fig. 7c), *ZNF598* knockdown or ZNF598^RING^ overexpression in inducible HEK293 cells (Extended Data Fig. 7d). In inducible hiPS cells, ZNF598^RING^ significantly increased ribosome pausing at 1,362 codons in 704 mRNAs (Fig. 5d and Supplementary Table 4), with a strong enrichment for AUG in the ribosomal P-site (Fig. 5e). The majority of these increased pauses (796; 58%) were within the first five codons of the respective CDS (hereafter start site pauses) and had Kozak consensus-like motifs (RCCAUGG)^85^ (Extended Data Fig. 7e). The remaining 566 increased internal pauses had the typical proline-rich internal pausing motif and no distinct nucleotide patterns (Extended Data Fig. 7f). By contrast, *ZNF598* knockdown in inducible hiPS cells resulted in only 331 codons with significantly increased pausing, which had nucleotide and amino acid motifs similar to those of internal pauses in wild-type hiPS cells (Fig. 5f,g and Supplementary Table 4). The differential pauses elicited by ZNF598 depletion or ZNF598^RING^ expression in inducible HEK293 cells were also not enriched for specific motifs (Extended Data Fig. 7g,h). These data suggest that ribosomes pause during initiation or early elongation when ZNF598-mediated ubiquitination is perturbed by RING domain mutations in human stem cells. The absence of these pauses upon ZNF598 depletion may be because of the global inhibition of translation initiation because of the ISR (Fig. 4e).

The 704 transcripts with start site pauses in ZNF598^RING^-expressing inducible hiPS cells were enriched for GO terms related to DNA packaging and mitochondrial proteins, which were not found in mRNAs with pauses in ZNF598-depleted cells (Fig. 5h,i). A total of 41 histone-encoding mRNAs had significantly increased ribosome pauses at or shortly after their annotated start sites, with little difference in footprint coverage downstream (Fig. 5j and Supplementary Table 4). The abundance of many of these histone mRNAs and other transcripts with start site pauses in inducible hiPS cells was significantly lower in inducible HEK293 cells (Extended Data Fig. 7i,j).

We reasoned that perturbed translation of histone mRNAs could be particularly problematic for stem cells, which progress more rapidly through the cell cycle and have a shortened G1 phase in comparison to somatic cells^86^. Canonical histone-encoding mRNAs are produced exclusively during the S phase^87^ and insufficient histones for packaging newly made DNA trigger S-phase arrest^88^. Both *ZNF598* knockdown and ZNF598^RING^ overexpression but not wild-type ZNF598 overexpression significantly increased the fraction of inducible hiPS cells in the S phase (Fig. 5k,l and Extended Data Fig. 7k). These data indicate that ZNF598-dependent ubiquitination is required for S-phase progression in human stem cells.

## ZNF598 detects ribosome collisions during initiation

**Fig. 6:**
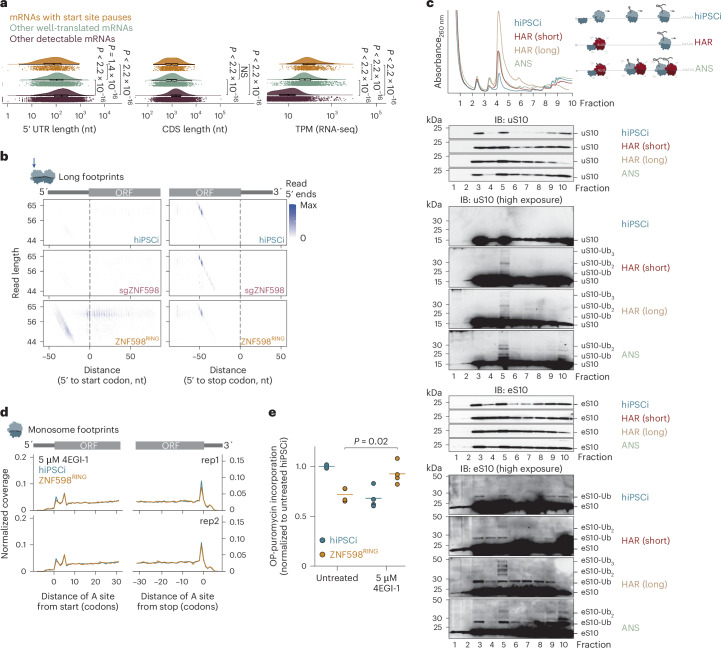
ZNF598 detects ribosome collisions during translation initiation. **a**, Comparison of 5′ UTR length, CDS length and transcript abundance (by TPM in RNA-seq) in mRNAs with significantly increased start site pauses (in the first five codons of the respective CDS) in ZNF598^RING^-expressing inducible hiPS cells (filtered for TPM > 1; *n* = 702), other well-translated mRNAs included in the pause site analysis (>0.5 footprints per codon, filtered for TPM > 1; *n* = 2,678) and remaining mRNAs with detectable expression in inducible hiPS cells (TPM > 1 in RNA-seq; *n* = 10,917). *P* values from a two-sided Wilcoxon test; NS, not significant (*P* > 0.01). Box plots: center line, median; box limits, upper and lower quartiles; whiskers, 1.5× the interquartile range. **b**, Representative density heatmaps of 42–68-nt footprints according to length and 5′ end position around CDS start (left) and stop (right) codons in control, *ZNF598* knockdown (sgZNF598) and ZNF598^RING^-expressing inducible hiPS cells (top to bottom) in one of two biological replicates. **c**, Polysome profiling (top) and immunoblot analysis of sucrose gradient fractions (bottom) from untreated inducible hiPS cells and after treatment with 0.05 mg L^−1^ ANS for 15 min or a short (2.5 min) or long (2 hours) treatment with 2 µg ml^−1^ HAR (*n* = 1 biological replicate from two independent experiments with similar results). **d**, Metagene profiles of ribosomal A sites from monosome footprints around CDS start and stop codons after treatment with 5 µM 4EGI-1 (*n* = 2 biological replicates). **e**, Global protein synthesis measurements by OPP labeling in control or ZNF598^RING^-expressing inducible hiPS cells before and after treatment with 5 µM 4EGI-1. Median fluorescence intensity quantified by flow cytometry (>10,000 cells per analysis) was normalized to values from untreated inducible hiPS cells (*n* = 4 biological replicates; *P* values from an unpaired two-tailed *t*-test). Source data

**Extended Data Fig. 8:**
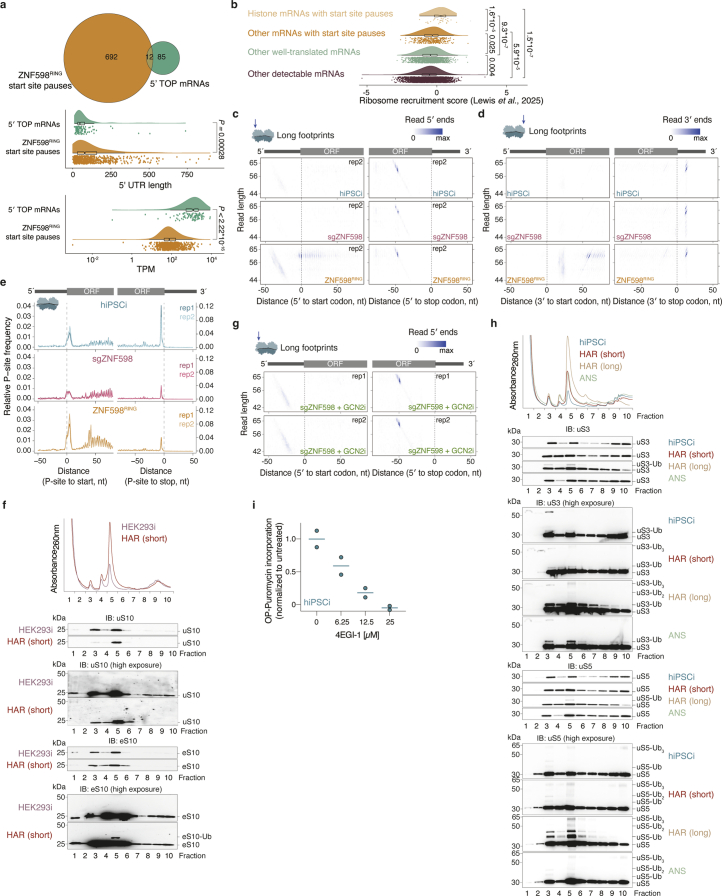
Analysis of long ribosome footprints and cellular responses to start site stalling. **a**, Venn diagram of overlaps between 5´ TOP motif mRNAs (*n* = 97) and transcripts with significantly increased A-site pauses in the first 5 codons in ZNF598^RING^-expressing hiPSCi (*n* = 704), and comparison of 5´ UTR lengths and TPM levels from RNA-Seq in both groups (*p*-value from two-sided Wilcoxon test). Box plots: center line, median; box limits, upper and lower quartiles; whiskers, 1.5× interquartile range. **b**, Comparison of ribosome recruitment scores for 5´ UTRs quantified by direct analysis of ribosome targeting (DART)^111^ for histone mRNAs with start site pauses (*n* = 29), other mRNAs with start site pauses (*n* = 351), other well-translated mRNAs (*n* = 1340), and other detectable mRNAs (*n* = 3791). Box plots: center line, median; box limits, upper and lower quartiles; whiskers, 1.5× the interquartile range. **c**, Density heatmaps of 50-80 nt ribosome footprints according to length and 5’ end position around CDS start (left) and stop sites (right) for a second biological replicate. **d**, Density heatmaps of 50-80 nt ribosome footprints according to length and 3’ end position around CDS start (left) and stop sites (right) for a second biological replicate. **e**, Metagene profiles of 50-80 nt ribosome footprints around CDS start and stop sites in hiPSCi (*n* = 2 biological replicates). **f**, Polysome profiles and immunoblot analysis of uS10 and eS10 in sucrose gradient fractions from control HEK293i or after a short (2.5 min) treatment with 2 µg/ml homoharringtonine (HAR) (*n* = 1 biological replicate). **g**, Metagene profiles of 50-80 nt ribosome footprints around CDS start and stop sites in ZNF598-depleted hiPSCi treated with GCN2 inhibitor (GCN2i, A-92, 1.25 µM*; n* = 2 biological replicates). **h**, Polysome profiles (from Fig. 6c) and immunoblot analysis of uS3 and uS5 in sucrose gradient fractions from control hiPSCi, after short (2.5 min) or long (2 hours) treatment with 2 µg/ml homoharringtonine, or after treatment with 0.05 mg/l anisomycin (ANS) for 15 minutes (*n* = 1 biological replicate). Membranes from Fig. 6c were stripped and re-probed with antibodies against uS3 and uS5. **i**, Global protein synthesis measurements by OPP labeling in control hiPSCi and/or after treatment with 6.25, 12.5, and 25 µM 4EGI-1 for 1 day. Median fluorescence intensity was quantified by flow cytometry ( > 10,000 cells/ analysis) and normalized to the average value in controls (*n* = 2 biological replicates). Source data

The mRNAs with increased start site pauses in ZNF598^RING^-expressing inducible hiPS cells had significantly shorter 5′ untranslated regions (UTRs) and higher abundance in comparison to other expressed mRNAs in inducible hiPS cells (*P* < 0.01; Fig. 6a). However, start site pauses did not increase on the majority (87%) of human mRNAs with 5′ terminal oligopolypyrimidine tracts (5′ TOP)^89,90^, which have extremely short 5′ UTRs^91–93^ (Extended Data Fig. 8a), suggesting that 5′ UTR length is not the sole determinant of increased ribosome pausing. Because ZNF598 mediates the specific ubiquitination of uS10 and eS10 residues at the 40S–40S interface of collided ribosomes^22,24,25^, we hypothesized that start site pauses could be because of a scanning 43S catching up with an initiating 80S on these messages. It was found shown that 43S recruitment (~10 s) and 60S joining (~30 s) on model substrates occur on a similar timescale to the transition of initiating ribosomes to elongation (~30 s), whereas the 43S scans the 5′ UTR at ~100 nt per second^94,95^. The potential for start site collisions would be particularly high for messages that are highly efficient in ribosome recruitment. When comparing ribosome recruitment scores for 5′ UTRs of human mRNAs^96^, we found that they were indeed significantly higher for mRNAs with start site pauses, particularly for histone mRNAs, than for transcripts without such pauses in ZNF598^RING^-expressing inducible hiPS cells (Extended Data Fig. 8b). These data suggest that start site pauses because of defective ubiquitination by ZNF598 occur on messages with highly efficient translation initiation.

We next asked whether we can detect 40S–80S collisions at translation start sites by ribosome profiling. The 43S complexes protect fragments of 20–60 nt from nuclease digestion^97,98^, while 80S diribosomes yield ~60-nt footprints^99,100^. We, therefore, analyzed 50–80-nt (long) ribosome footprints from control inducible hiPS cells and upon ZNF598^RING^ expression or *ZNF598* knockdown. We detected ~60-nt footprints with 5′ ends mapping ~50 nt from annotated stop codons in all conditions (Fig. 6b and Extended Data Fig. 8c), consistent with 80S ribosome collisions during translation termination^80,100^. An additional density of footprints 45–62 nt in length was detectable in ZNF598^RING^-expressing inducible hiPS cells. The 5′ ends of these footprints mapped 25–40 nt upstream of annotated start codons and their 3′ ends mapped ~18 nt downstream. Footprints with a length of ~60-nt with 5′ ends around annotated start sites, indicative of 80S–80S collisions during early elongation, were also detectable only in ZNF598^RING^-expressing inducible hiPS cells (Fig. 6b and Extended Data Fig 8c–e). Together, these data suggest that ZNF598^RING^ stabilizes 40S–80S collisions during initiation and 80S–80S collisions during early rounds of elongation in inducible hiPS cells.

To test whether increased potential for ribosome collisions during initiation can lead to ZNF598-mediated uS10 and eS10 ubiquitination, we exposed inducible hiPS cells to two translation inhibitors: homoharringtonine (HAR), which blocks the first peptide bond formation^101^, and anisomycin (ANS), which, at intermediate concentrations, induces collisions of elongating ribosomes that trigger p38 phosphorylation^50^. Both drugs could result in 40S–80S collisions as they do not inhibit 40S loading onto mRNA. Brief (2.5 min) or extended (2 h) HAR treatment of inducible hiPS cells led to polysomal collapse and a concurrent increase in the 80S peak, indicating ribosome runoff (Fig. 6c). In line with our predictions, we detected uS10 polyubiquitination in 80S fractions from inducible hiPS cells treated with both inhibitors, as well as in heavy polysome fractions from cells treated with ANS under conditions that induce p38 phosphorylation (Figs. 6c and 4g). Monoubiquitinated eS10, which is not sufficient to trigger ribosome subunit dissociation^60^, was present in 40S, 80S and heavy polysome fractions in untreated inducible hiPS cells. However, eS10 polyubiquitination increased specifically in 80S fractions upon treatment with both drugs, as well as in heavy polysome fractions in ANS (Fig. 6c). In inducible HEK293 cells, by contrast, a 2.5-min HAR treatment did not lead to uS10 ubiquitination and only mildly increased eS10 monoubiquitination (Extended Data Fig. 8f). Additionally, pharmacological inhibition of GCN2 in ZNF598-depleted inducible hiPS cells increased the density of long footprints with 5′ ends mapping 25–40 nt upstream of start codons (Extended Data Fig. 8g). Collectively, these data suggest that ZNF598 ubiquitinates uS10 and eS10 on ribosomes colliding at translation start sites in human stem cells. This pathway is distinct from the initiation ribosome quality control, in which RNF10 ubiquitinates uS3 and uS5 to flag ribosomes terminally stalled during initiation^102–106^. Consistently, uS3 and uS5 ubiquitination increased substantially in inducible hiPS cells upon prolonged but not brief HAR exposure (Extended Data Fig. 8h), suggesting that ZNF598 detects start site ribosome collisions in early or mild stress conditions.

**Fig. 7:**
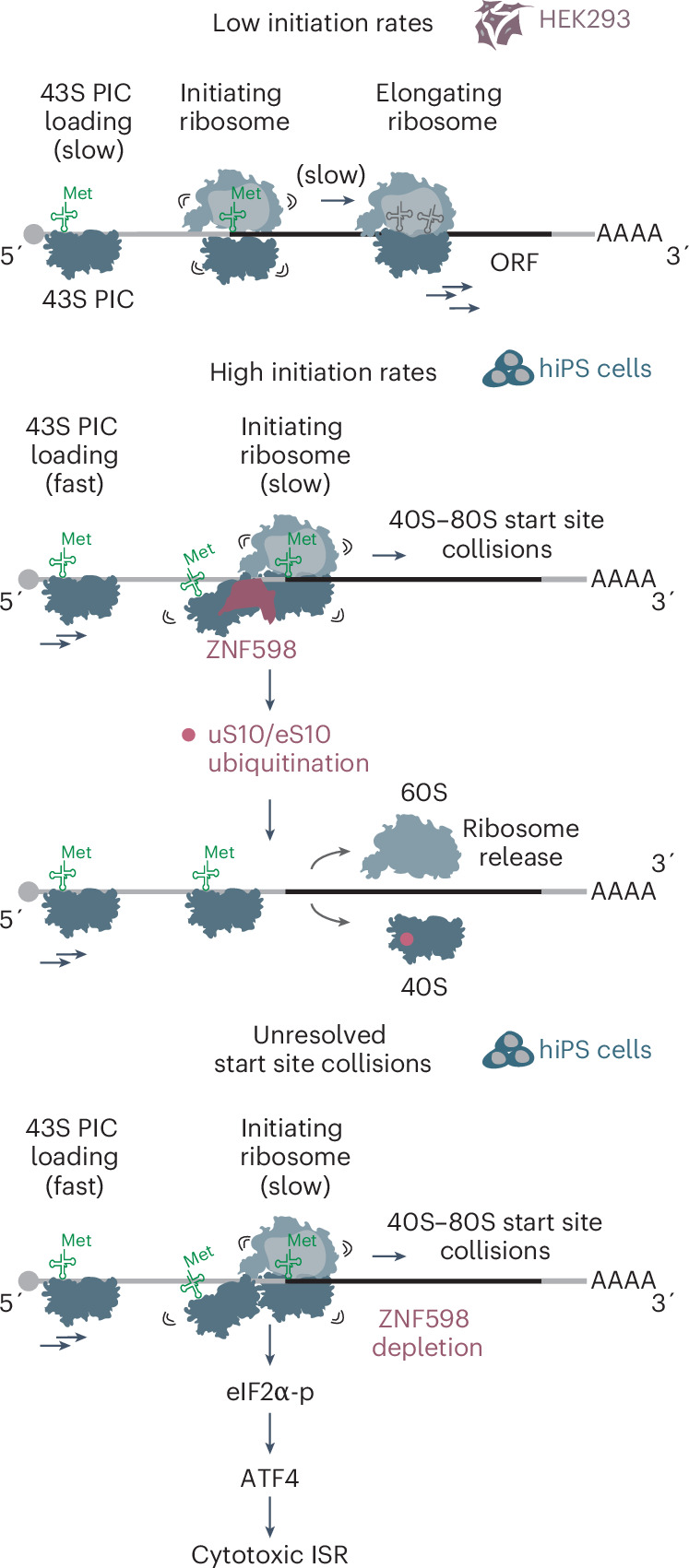
Model for ZNF598-mediated surveillance of translation initiation. ZNF598 prevents a cytotoxic ISR in human stem cells triggered by start site collisions.

If ZNF598 detects a scanning 43S collided with an 80S ribosome that is transitioning to elongation, reducing 43S loading on mRNAs should decrease the potential for such collisions. To test this, we treated inducible hiPS cells with 4EGI-1, an inhibitor of eIF4E–eIF4G complex formation^107^, which we used at a concentration that only minimally reduced global protein synthesis (by ~25%; Extended Data Fig. 8i). This was sufficient to abolish excess ribosome density at start sites and rescue the global translation defects of ZNF598^RING^-expressing inducible hiPS cells (Fig. 6d,e). Taken together, our data suggest a model in which ZNF598 detects collisions of scanning 43S with 80S ribosomes at start codons on mRNAs with high initiation rates (Fig. 7).

## Discussion

By mapping the essentiality of mRNA translation-related genes across diverse hiPS cell-derived cell types, our study provides a rich resource for dissecting pathway-specific regulatory mechanisms in physiologically relevant settings. Through optimized CRISPRi screening and differentiation workflows, we achieved robust and efficient gene knockdown in dividing human cells (hiPS cells and NPCs) and in postmitotic hiPS cell-derived neuronal and cardiac cells. This comparative approach enabled us to identify core and cell-type-specific genetic dependencies and discover that human stem cells express mRNAs with highly efficient translation initiation that require the ribosome collision sensor ZNF598 to prevent a cytotoxic ISR.

Activation of the ISR, which also happens upon deletion of the *ZNF598* homolog *HEL2* in yeast, may decrease ribosomal flux along messages, limiting the chances for ribosome collisions^80^. Decreased ribosome flux may, however, mask the endogenous translation events that require surveillance, explaining why their discovery has been so challenging. The common activation of the ISR upon deletion or repression of distinct ribosome quality control genes^77–79^ but not upon expression of their corresponding catalytically inactive mutants^81^ suggests that the broader use of such mutants may be better suited for identifying the physiological triggers of these pathways in cells. Our data also indicate that a higher basal level of ISR activation in HEK293 and potentially other immortalized cell lines could contribute to their resilience to ribosome rescue perturbations^23^.

By identifying the essential role of ZNF598 in human stem cells and profiling the consequences of its catalytic inactivation, we discovered a previously unappreciated source of problematic translation events: the slow transition of initiating ribosomes to elongation. This transition is two orders of magnitude slower than subsequent elongation rounds^94,95^ and our data suggest that ZNF598 detects queuing and collisions that occur if a scanning 43S catches up with an initiating 80S. Furthermore, 43S–80S collisions were recently shown to occur when decoding-incompetent 80S ribosomes with 18S ribosomal RNA (rRNA) defects stall at start sites in *GCN2-*knockout human cells^106^. Structures of ZNF598 are still lacking but uS10 and eS10, which are targeted by ZNF598, reside at the 40S–40S interface in collided diribosomes^25,52,108^, supporting the notion that the surface recognized by ZNF598 could be formed by a 40S–80S collision. Mechanisms to resolve such collisions would ensure the accuracy and fidelity of start site selection by preventing frameshifting of the initiating 80S (ref. ^109^). Our data indicate that such collisions may occur only on some mRNAs or in specific cell contexts and suggest that higher basal eIF2α phosphorylation levels might limit them by decreasing ribosome loading in some cell lines.

We find that start site collisions in human stem cells occur in a subset of mRNAs with initiation sites in a strong Kozak context, shorter than average 5′ UTRs and with high ribosomal loading rates^96^. Poignant examples of such transcripts are those encoding histone proteins, which are synthesized in large amounts only during the S phase. Ribosomes are loaded on newly made histone mRNAs approximately five times more rapidly than on housekeeping mRNAs in mouse ES cells^110^. Such rapid loading would enable cells to quickly produce large amounts of protein from these messages but may also increase the probability of 40S–80S collisions at start sites. Interestingly, we did not detect start site pauses in most 5′ TOP mRNAs, which have extremely short 5′ UTRs that may not be compatible with the recruitment of 43S before initiating ribosomes have cleared the AUG^111^. Extremely short 5′ UTRs could, thus, have evolved to protect messages from start site ribosome collisions.

While depletion of most translation quality control factors did not notably impact the survival of neurons and CMs in our screens, future studies assessing cellular function, morphology or physiological activity will be necessary to fully define their contributions in these cell types. The silencing of mRNA translation regulators can also have pleiotropic effects on cell physiology. Our discovery of ZNF598’s role in detecting ribosome collisions during translation initiation in human stem cells emphasizes the need to study ribosome quality control in biological contexts where it is necessary to maintain the translational homeostasis of endogenous mRNAs.

## Methods

### Cell culture and inducible hiPS cell differentiation

To generate the inducible hiPS cell line, the reference HPSI0214i *kucg-2* cell line^29^ was engineered to express KRAB–dCas9 from a *TRE3G* promoter at the *AAVS1* locus using a previously established workflow^27,30^. The inducible HEK293 cell line was constructed in a similar fashion^112^. Inducible hiPS cells were cultured in mTeSR Plus on Matrigel-coated plates at 37 °C in 5% CO_2_. The medium was exchanged every other day and cells were propagated as clusters with 0.5 mM EDTA–PBS every 5 days in a ratio of 1:20. For experiments, cells were singularized with Accutase and resuspended in mTeSR Plus containing 10 µM of the ROCK inhibitor Y-27632. Viable cells were counted with Trypan blue in the Cell Countess II system and pelleted for 5 min at 200*g*. Cells were seeded in mTeSR Plus with 10 µM Y-27632 and the medium was exchanged with mTeSR Plus without Y-27632 on the next day.

The inducible hiPS cell line was differentiated into NPCs using small molecules with previously established protocols^30,113,114^ in the continuous presence of 2 µM doxycycline (Sigma-Aldrich, D3447) to prevent *TRE3G* promoter silencing. Briefly, inducible hiPS cells were cultured to 90% confluency and dissociated by scratching a checkered pattern into the dish, followed by incubation with collagenase IV for 10–15 min at 37 °C. Cell clusters were scratched off the plate and pelleted by gravity in a 15-ml tube containing N2B27 neurobasal medium (Gibco, 21103049): DMEM/F12 (Gibco, 2133) 50:50, 0.5× N2 (Thermo Fisher Scientific, 17502048), 0.5× B27 (Thermo Fisher Scientific, 12587010) and 2 mM GlutaMAX (Gibco, 35050061). For embryoid body formation, cell clusters were washed once with N2B27 medium and transferred into NPC induction medium (NPC-IM; N2B27 with 200 µM ascorbic acid (AA; Sigma-Aldrich, A4403), 3 µM CHIR99021 (Axon Medchem, Axon1386), 0.5 µM phorbol 12-myristate 13-acetate (PMA; Santa Cruz Biotechnology, sc-202785A), 150 nM dorsomorphin (Absource, S7306) and 10 µM SB431542 (Biomol, Cay12031)) with 5 µM Y-27632 in a sterile uncoated dish and incubated at 37 °C in 5% CO_2_. The medium was exchanged the next day to remove Y-27632 and embryoid bodies were maintained in NPC-IM for 6 days with medium exchanges every day. Embryoid bodies were then dissociated by pipetting and plated into Matrigel-coated wells in NPC culture medium (N2B27 with 200 µM AA, 3 µM CHIR99021 and 0.5 µM PMA). The medium was exchanged every day. The first rounds of passaging were performed in a sequential digest to purify the NPCs from other cell types. After derivation, NPCs were passaged as single cells using Accutase every 5 days in a ratio of 1:10.

For neuron derivation from NPCs^115,30^, cells were dissociated with Accutase and half a million cells were maintained in a 12-well containing patterning medium for 6 days (N2B27 with 200 µM AA, 1 µM retinoic acid (Sigma-Aldrich, R2625), 0.5 µM PMA and 10 ng ml^−1^ glial cell-line-derived and brain-derived neurotrophic factors (GDNF/BDNF; Peprotech, 450-10 and 450-02)). Consequently, medium was changed to maturation medium (N2B27 with 200 µM AA, 100 µM dibutyryl cyclic AMP (Sigma-Aldrich, D0627), 5 ng ml^−1^ GDNF/BDNF and 1 ng ml^−1^ tumor growth factor β3 (Peprotech, AF-100-36E)). Next, 5 ng µl^−1^ activin A (Life Technologies, PHG9014) was added for the first 2 days of maturation. Cells were cultured for another 10 days, changing the medium every day. To further stimulate neuron maturation, 0.1 µM CompE (Merck, 565790) was added for 2 days during this period. After a total of 16 days, cells were singularized with Accutase, resuspended in maturation medium, pelleted by centrifugation for 5 min at 200*g* and reseeded to a new plate. Experiments were performed on day 21.

CM differentiation from inducible hiPS cells was performed as previously described^114,30^. Briefly, cells were singularized with Accutase and seeded in differentiation medium with 10 µM Y-27632 (KO-DMEM (Gibco, 10829-018), 2 mM l-glutamine (Gibco, 25030-024), 5 µg ml^−1^ each of insulin, transferrin and selenious acid (Corning, 354351), 10 ng ml^−1^ fibroblast growth factor 2 (Peprotech, 100-18B-250), 1 µM CHIR99201 (Axon Medchem, Axon1386), 1 ng ml^−1^ bone morphogenetic protein 4 (R&D, 314-BP-010) and 5 ng ml^−1^ activin A (Life Technologies, PHG9014)) on Matrigel-coated plates. After 24 h, medium was exchanged to transferrin and selenium (TS) medium (KO-DMEM, 2 mM glutamine, 5.5 µg ml^−1^ human transferrin (Sigma-Aldrich, TS8158), 6.7 ng ml^−1^ sodium selenite (Sigma-Aldrich, 214485) and 250 µM AA). Cells were maintained in TS medium for a total of 9 days with daily medium exchange and 0.2 µM Wnt inhibitor C59 (Tocris, 5148) was added on days 2 and 3. For CM enrichment, cells were cultured in medium containing lactic acid on day 10 (DMEM without glucose (Gibco, A13320-01), 2 mM glutamine, 5.5 µg ml^−1^ human transferrin, 6.7 ng µl^−1^ sodium selenite, 250 µM AA and 4 mM lactic acid (Sigma-Aldrich, L4263-100ML)^116^. Cells were then singularized with Accutase, plated in CM maturation medium (KO-DMEM, fetal calf serum (FCS) 2% (Gibco, 16000-044) and 1:100 glutamine) on Matrigel-coated wells and maintained for another 5 days before harvesting.

Inducible HEK293 cells and Lenti-X 293T cells (Takara, 632180) were cultured in DMEM high-glucose medium supplemented with 10% FCS at 37 °C in 5% CO_2_ and passaged using 0.25% trypsin–EDTA every other day in a ratio of 1:10 to 1:20.

### CRISPRi library design

An adapted version of the CRISPRiaDesign workflow^31^ (https://github.com/mhorlbeck/CRISPRiaDesign) was used to design sgRNAs to target the transcription start site (TSS) of selected genes as previously described^30^. The sgRNAs with highest predicted activity scores and no predicted off-target effects were used for sgRNA library construction.

### Pooled CRISPRi screens

The sgRNAs for the CRISPRi screens were ordered as an oligonucleotide pool at Twist Bioscience with matching overhangs for a parental vector based on pU6-sgRNA-EF1Alpha-puro-T2A-BFP (Addgene, plasmid 60955; a gift from J. Weissman), in which BFP was exchanged with mKate. The sgRNA oligonucleotide pool was amplified using KAPA HiFi Hotstart polymerase with the high-fidelity buffer (95 °C for 3 min, followed by 12 cycles of 98 °C for 20 s, 56 °C for 15 s and 72 °C for 15 s and a final elongation at 72 °C for 1 min) and cleaned up using the Zymo DNA clean and concentrator kit (Zymo Research, D4003). The parental vector was digested with BstXI and BlpI. Gibson assembly reactions were performed in a 3:1 ratio of insert to vector and transformed into MegaX competent cells. Colonies were scraped off plates and the plasmid DNA was extracted using the Plasmid Plus Midi kit (Qiagen, 12943). For lentivirus production, Lenti-X 293T cells were cotransfected with the sgRNA plasmid pool and third-generation lentiviral packaging plasmid mix (4:1:1, pMDLg/pRRE (Addgene, plasmid 12251), pRSV-Rev (Addgene, plasmid 12253) and pMD2.G (Addgene, plasmid 12259; all gifts from D. Trono) in a 1:1 ratio with TransIT-Lenti transfection reagent (Mirus, MIR6603) following the manufacturer’s instructions. After 2 days, the viral supernatant was collected, filtered through a 0.45-μm PVDF syringe filter and precipitated with lentivirus precipitation solution (Alstembio, VC125) at 4 °C overnight. Lentivirus stocks were resuspended in cold PBS and stored at −80 °C.

Growth screens were performed on dividing cells (inducible hiPS cells, inducible HEK293 cells and NPCs) in three biological replicates. Inducible hiPS cells were transduced by coseeding cells and lentiviral stocks at the same time with an initial transduction rate of 30% based on the mKate signal. Cells were reseeded after 2 days in medium containing 2 μg ml^−1^ puromycin and selected for two passages. After 3 days of recovery without puromycin, 3 × 10^6^ cells representing a 1,000× coverage of the sgRNA library were seeded with and without 2 μM doxycycline and passaged every 5 days. Inducible hiPS cells were harvested after 10 days, which corresponds to approximately ten cell doubling times. For inducible HEK293 cells, 8 μg of TransduceIT (Mirus, MIR6620) was added during transduction and cells were selected in 1 μg ml^−1^ puromycin. Transduced inducible HEK293 cells were passaged every 3 days during the screens and harvested after 12 days, which corresponds to approximately ten doublings. A total of 8 × 10^6^ NPCs were transduced in the presence of 2 μM doxycycline, reseeded in 2.5 μg ml^−1^ puromycin the next day and selected for 2 days. The culture was then split in two and 3 × 10^6^ cells were seeded either with or without 2 μM doxycycline. NPCs were passaged every 5 days and harvested after 15 days, which corresponds to approximately ten doublings.

Differentiation screens were performed by adding 2 μM doxycycline to induce KRAB–dCas9 expression at the start of the derivation of CMs or NPCs from inducible hiPS cells and of neurons from NPCs. For survival screens, lentiviral transductions were performed in two biological replicates for CMs and three biological replicates for neurons. For CMs, inducible hiPS cells were transduced and selected with puromycin as for the growth screens and CM derivation was initialized after puromycin selection. CMs were split at the reseeding step to two wells either without or with 2 μM doxycycline and cultured for an additional 20 days before harvesting. For neurons, NPCs were transduced and selected with puromycin as for growth screens and neuron derivation was initialized after puromycin selection. Neurons were split at the reseeding step to two wells either without or with 2 μM doxycycline and cultured for an additional 20 days before harvesting.

For library preparation, 5 × 10^6^ cells were harvested per screen and genomic DNA was extracted using the Nucleospin blood kit (Macherey&Nagel, 740951.50). Then, 20 μg of DNA was amplified using NEBNext Ultra II Q5 master mix. Two custom primer sets were used to maximize read variety during sequencing (https://weissman.wi.mit.edu/resources/IlluminaSequencingSamplePrep_old.pdf; Supplementary Table 5). Each PCR reaction contained 5 μg of DNA and was amplified with the following settings: 98 °C for 2 min, followed by 22 cycles of 98 °C for 10 s, 60 °C for 30 s and 65 °C for 45 s, with a final elongation at 65 °C for 5 min. PCR reactions for the same sample were pooled and concentrated using the Zymo DNA clean and concentrator kit (Zymo Research, D4003). The reactions were run on an 8% polyacrylamide and 1× TBE gel, and the PCR product corresponding to the sgRNA library was excised. The gel slices were crushed with a disposable pestle and DNA was eluted in water overnight at room temperature on a rotating wheel. The next day, gel debris was removed by a Spin-X filter and library DNA was recovered by ethanol precipitation. The final library concentration was measured with the Qubit dsDNA high-sensitivity (HS) kit. Libraries were sequenced for 50 cycles on a NextSeq 500 platform (Illumina).

### Single and dual sgRNA knockdown experiments

**Extended Data Fig. 9:**
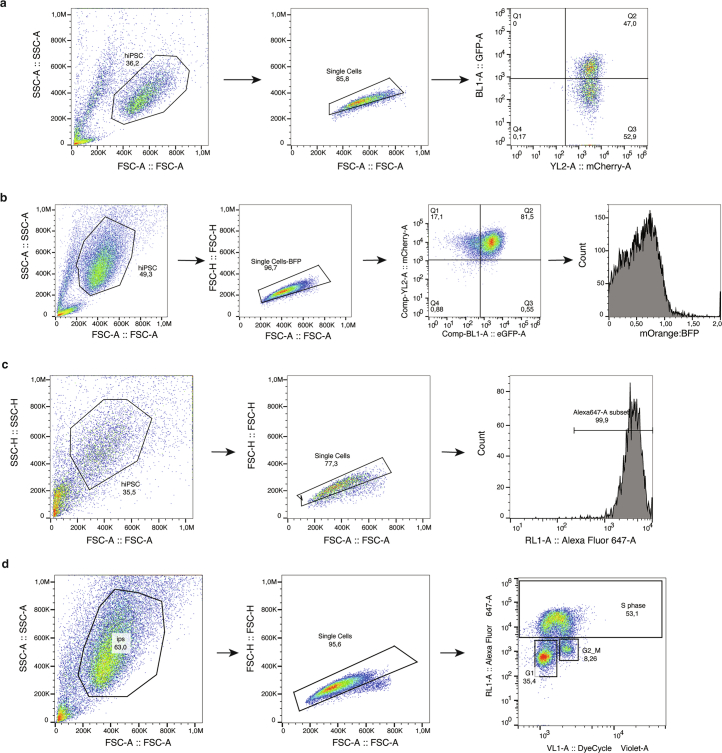
Gating strategies for flow cytometry. **a**, Representative gating strategy for growth assays (Figs. 3b, 4e, 4f and Extended Data Fig. 2c, 2e, 4a, 4b, 4c, 4d, 5a). **b**, Representative gating strategy for stalling reporter assays (Fig. 3c,d,e and Extended Data Fig. 6b). **c**, Representative gating strategy for OPP assays, phospho-staining, and mCherry expression assays (Figs. 4a, 6e and Extended Data Figs. 1c, 1g, 1h, 5e, 5f, 6g, 8i). **d**, Representative gating strategy for EdU assays (Figs. 5k, 5l and Extended Data Fig. 7k).

For knockdown experiments with individual sgRNAs, the parental library lentiviral vector was first modified by exchanging mKate with GFP to generate mU6-sgRNA-EF1A-Puro-GFP, followed by cloning individual sgRNAs (listed in Supplementary Table 6) ordered as oligonucleotides with matching overhangs by Gibson assembly^30^. For combinatorial gene knockdown, the individual sgRNA lentiviral vector was first modified by removing SapI sites, followed by insertion of the dual sgRNA cassette from pKLV2.2-h7SKgRNA5(SapI)-hU6gRNA5(BbsI)-PGKpuroBFP-W^117^ (Addgene, plasmid 72666; a gift from from K. Yusa) by Gibson assembly. Lentiviral transduction was performed as for the pooled screens. For growth assays, the cell population was split into two wells (with and without 2 µM doxycycline) and cultured for a time period of 20 population doublings. Cells were passaged every 4 days and the percentage of GFP^+^ cells was measured on the Attune NxT system. For follow-up analysis, inducible hiPS cells and inducible HEK293 cells were selected with 2 or 1 μg ml^−1^ puromycin for 3–5 days until the fraction of GFP^+^ cells exceeded 80%. NPC were selected with 2.5 μg ml^−1^ puromycin ± 2 µM doxycycline, respectively, until >80% cells were GFP^+^. Gating strategies for flow cytometry are represented in Extended Data Fig. 9.

### Stalling readthrough reporter experiments

Previously described reporter constructs^24,55^ were adapted by exchanging the cytomegalovirus promoter with a UbC promoter, replacing fluorescent cassettes (GFP to BFP and red fluorescent protein (RFP) to mOrange) and exchanging the first 2A skipping sequence to T2A. For this, a UbC-BFP-T2A-FLAG-XBP1stall-P2A-mOrange cassette^55^ was synthesized by Twist Bioscience and inserted into a plasmid containing sequences for lentiviral packaging (Addgene, 60955). The XBP1 sequence was removed to yield the ‘no-stall’ reporter or replaced with (AAA)_20_ derived from pmGFP-P2A-K(AAA)20-P2A-RFP (Addgene, plasmid 105688; a gift from R. Hegde). For stalling readthrough assays, puromycin-selected cells transduced with individual sgRNA expression vectors (mU6-sgRNA-EF1A-Puro-GFP) were cultured with 2 µM doxycycline for 3 days, transduced with the stalling constructs and allowed to recover for 2 days before analysis. Cells were harvested and fluorescence was analyzed on an Attune NxT flow cytometer. Fluorescent signals were compensated by analyzing cells expressing each fluorophore by itself expressed from the same promoter. The median fluorescence intensity of samples expressing a stalling construct were normalized to the corresponding no-stall control construct under the same knockdown condition.

### ZNF598^RING^ and wild-type ZNF598 expression

The cDNA for wild-type ZNF598 or the ZNF598^RING^ C29S;C32S mutant^54^ with a C-terminal HA tag was synthesized by Twist Bioscience as an A2UCOE-EF1a-ZNF598-2A-BLC cassette and inserted into a plasmid containing sequences for lentiviral packaging (Addgene, 60955). Cells were transduced with the expression constructs and cultured for a total of 2 days before harvesting. For reduced ribosome loading experiments in inducible hiPS cells, 5 µM 4EGI-1 (Sigma-Aldrich, 324517) was added for 18 h before harvesting.

### Growth complementation assays of HBS1L-depleted cells

The cDNA for wild-type HBS1L or the HBS1L H348A GTPase-inactive mutant^118–120^ was synthesized as a fusion to eBFP separated by a 2A ribosome skipping sequence at Twist Bioscience. The resulting expression cassettes were inserted downstream of a UbC promoter in pLJC6-EV (Addgene, plasmid 163454; a gift from J. Cantor). Cells transduced with an sgRNA targeting *HBS1L* were selected with puromycin to obtain ~70% GFP^+^ cells. Subsequently, cells were transduced with an HBS1L expression vector and cultured with or without 2 µM doxycycline for 8 days. Cells were gated for BFP^+^ cells and the percentage of GFP^+^ cells was measured on an Attune NxT flow cytometer.

### Global protein synthesis measurements

Puromycin-selected cells transduced with mU6-sgRNA-EF1A-Puro-GFP lentiviral vectors were washed twice with PBS and incubated in methionine-free DMEM supplemented with 10% dialyzed FCS (Thermo Fisher Scientific, A3382001) for 30 min and incubated in medium containing 2 µM l-homopropargylglycine (HPG; Jena Bioscience, CLK-1067) for 30 min. Control and ZNF598^RING^-expressing inducible hiPS cells were labeled by the addition of 20 µM *O*-propargyl-puromycin (OPP; Thermo Fisher Scientific, C10459) to the culture medium for 30 min. Cells were fixed for 10 min in 3.7% formaldehyde in TBS, washed with TBS and permeabilized for 15 min in TBS-T (0.5% Tween-20). HPG and OPP were detected by click chemistry following a 30-min incubation in 100 mM Tris pH 8, 1 mM CuSO_4_, 20 µM AF647-picolyl-azide (Jena Bioscience, CLK-1300) and 100 mM AA. Cells were washed three times in TBS-T (0.2% Tween-20) and fluorescence was analyzed on an Attune NxT flow cytometer.

### LDH assays

Puromycin-selected cells transduced with mU6-sgRNA-EF1A-Puro-GFP lentiviral vectors were cultured with 2 µM doxycycline until onset of growth defects in inducible hiPS cells (~75% of viable cells). The supernatant was collected to assess cellular LDH levels the using CytoTox 96 nonradioactive cytotoxicity assay kit (Promega, G1780) according to the manufacturer’s instructions.

### Cell-cycle analysis by 5-ethynyl-2′-deoxyuridine (EdU) staining

Culture medium was exchanged with fresh medium 2 h before harvesting. Cells were labeled using the Click-iT Plus EdU Alexa Fluor 647 Flow Cytometry Assay Kit (Thermo Fisher Scientific, C10634) according to the manufacturer’s instructions. In brief, cells were incubated with 10 µM EdU for 1 h and collected by trypsinization. Cells were washed once in 1% BSA–PBS and fixed for 15 min. Cells were washed once in 1% BSA–PBS and permeabilized for 15 min before the click reaction. Cells were incubated for 30 min in the dark and washed twice. Following FxCycle dye addition (Thermo Fisher Scientific, F10347), cells were incubated for 30 min in the dark and fluorescence intensity was measured on an Attune NxT flow cytometer.

### RNA isolation

Culture medium was aspirated and cells were directly lysed in the culture plates by adding LiDS–LET buffer (5% LiDS in 20 mM Tris, 100 mM LiCl, 2 mM EDTA and 5 mM DTT, pH 7.4, supplemented with 100 μg ml^−1^ proteinase K). Lysates were collected, incubated at 60 °C for 10 min, passed ten times through a 26G needle and mixed by vortexing. RNA was extracted by the addition of two volumes of cold acid phenol (pH 4.3), one tenth volume of 1-bromo-3-chloropropane (BCP) and 50 µg of glycogen (Thermo Fisher Scientific, AM9510). Following centrifugation for 5 min at 10,000*g* at 4 °C, the aqueous phase was transferred to a new tube and a second phenol–BCP extraction was performed. RNA was precipitated with by the addition of 2.5 volumes of ethanol at −20 °C for 30 min. After 30 min of centrifugation at 16,000*g* at 4 °C, cell pellets were air-dried and resuspended in RNase-free water. RNA concentration was measured by Nanodrop and samples were stored at −80 °C.

### RT–qPCR

First, 5 μg of total RNA were treated with 2 U of Turbo DNase (Thermo Fisher Scientific, AM2238) for 30 min at 37 °C at 1,500 rpm. The RNA was then cleaned up by phenol–BCP extraction. For RT, 1 μg of DNase-treated RNA was reverse-transcribed using the Protoscript II first-strand cDNA synthesis kit (New England Biolabs (NEB), E6560). RT–qPCR was performed with the KAPA SYBR fast qPCR mix (Roche, KK4601) and a 1:80 dilution of the cDNA. For analysis, ΔΔ*C*_*t*_ values were calculated relative to a nontargeting control. qPCR primer efficiency was tested with a serial dilution; only primer pairs with an efficiency of 1.9–2.1 were used for experiments.

### Immunoblotting

Cells were quickly rinsed with cold PBS and lysed by the addition of 20 mM Tris pH 7.5, 150 mM NaCl, 1% NP-40, 0.5% sodium deoxycholate and 0.1% SDS supplemented with 10 µg ml^−1^ aprotinin, 20 µM leupeptin, 2.5 µM pepstatin A, 0.5 mM AEBSF and 1× phosphatase inhibitor cocktail (Cell Signaling, 5870) to culture plates. Extracts were collected and incubated on ice for 20 minutes. Insoluble fractions were pelleted by 5-min centrifugation at 10,000*g* at 4 °C. EDTA was added to a final concentration of 5 mM to the supernatants and protein concentration was then quantified with the Pierce BCA protein assay kit (Thermo Fisher Scientific, 23225). Then, 15 μg of total protein from each sample was resolved on 4–12% Bis–Tris precast polyacrylamide gels (Life Technologies) in Bolt MOPS SDS running buffer (Invitrogen, B0001). Proteins were then transferred to a 0.2 μM nitrocellulose membrane (Amersham, 10600015) for 30–40 min at 25 V using the semidry TransBlot Turbo system (Biorad). For total protein visualization, the membranes were stained with Ponceau (0.5% Ponceau S and 1% acetic acid) for 3 min at room temperature, rinsed with distilled water and imaged on a BioRad GelDoc Imager. Membranes were destained with PBST (0.1% Tween-20), blocked for 1 h in 5% milk in PBST (0.1% Tween-20) and further incubated with primary antibodies in blocking solution overnight at 4 °C (ZNF598: 1:1,000, Abcam, ab135921; PELO F-4: 1:1,000, Santa Cruz Biotechnology, sc-393418; HBS1L: 1:1,000, Atlas Antibodies, HPA029729; ASCC3: 1:1,000, Bethyl Laboratories, A304-015A; eIF2α: 1:1,000, Cell Signaling, 9722; eIF2α-p S51: 1:1,000, Abcam, ab32157; p38: 1:1,000, Cell Signaling, 9212; p38-p T180/Y182: 1:1,000, Cell Signaling, 9211; eS10: 1:1,000, Abcam, ab151550; uS10: 1:1,000, Abcam, ab133776; uS5: 1:1,000, Bethyl Laboratories, A303-794A; uS3: 1:1,000, Bethyl Laboratories, A303-840A). The next day, membranes were washed in PBST (0.1% Tween-20) and incubated with horseradish peroxidase (HRP)-labeled secondary antibodies in 5% milk in PBST (0.1% Tween-20) at room temperature for 1 h (anti-rabbit IgG–HRP: 1:4,000, Dianova, 111-035-003; anti-mouse IgG–HRP: 1:4,000, Dianova, 115-035-003). Membranes were incubated for 5 min in SuperSignal West Pico PLUS (Thermo Fisher Scientific, 34577) and proteins were visualized on an iBright system (Thermo Fisher Scientific).

For comparisons of basal stress activation in inducible hiPS cells and inducible HEK293 cells, membranes stained with eIF2α-p and p38-p antibodies were stripped using Restore Western Blot stripping buffer (Thermo Fisher Scientific, 21059) for 30 min at room temperature, blocked for 1 h and reprobed with eIF2α and p38 antibodies, respectively. For polysome protein content analyses, membranes stained with eS10 and uS10 antibodies were stripped and reprobed with uS5 and uS3 antibodies, respectively.

### Immunostaining

Cells were grown on glass-bottom dishes (ibidi, 80827) and fixed in 3.7% formaldehyde for 10 min at room temperature. Formaldehyde was stepwise replaced with PBST (0.02% Tween-20). Inducible hiPS cells and NPCs were then permeabilized for 10 min with 0.5% Triton X-100 in PBST (0.02% Tween-20) and blocked for 1 h in blocking solution (3% BSA and 0.1% Triton X-100 in PBS). Cells were incubated overnight with the primary antibody diluted in blocking solution at 4 °C (POU5F1 C-10: 1:400, SCBT, sc-5279; NANOG P1-2D8: 1:200, Millipore, MABD24; PAX6: 1:200, Abcam, ab5790; NES: 1:200, R&D Systems, MAB1259). Cells were washed three times in PBST (0.02% Tween-20) and incubated with 500 ng of DAPI (Roche, 10236276001) and the secondary antibody diluted in blocking solution for 1 h at room temperature (goat anti-mouse Alexa Fluor 488: 1:2,000, Thermo Fisher Scientific, A-11001; goat anti-rabbit Alexa Fluor 488: 1:2,000, Thermo Fisher Scientific, A-11034; goat anti-mouse Alexa Fluor 633: 1:500, Thermo Fisher Scientific, A-21052). Cells were washed three times in PBST (0.02% Tween-20) before imaging. Neurons were permeabilized for 10 min in PBST (0.7% Tween-20) and blocked for 1 h in blocking solution (1% BSA, 0.1% Triton X-100 and 10% FCS in PBS). Cells were washed once in 0.1% BSA in PBS and incubated overnight with the primary antibody diluted in 1% BSA in PBS at 4 °C (MAP2: 1:1,000, Abcam, ab92434; CHAT: 1:200, Abcam, ab6168). Cells were washed three times in 0.1% BSA in PBST (0.05% Tween) and incubated with 500 ng of DAPI and the secondary antibody (goat anti-rabbit A633: 1,500, Thermo Fisher Scientific, A21070; goat anti-chicken A488: 1:2,000, Thermo Fisher Scientific, A-11039) diluted in 1% BSA in PBS for 1 h at room temperature. Cells were washed three times in 0.1% BSA in PBST (0.05% Tween) before imaging. CMs were blocked and permeabilized in 3% BSA and 0.1% Triton X-100 in PBS for 1 h at room temperature. Cells were washed three times with PBST (0.1% Tween-20) and incubated with primary antibody (anti-ACTN2: 1:800, Sigma-Aldrich, A7811; anti-cTNT CT3: 1:5, deposited to the DSHB by J.J.-C. Lin) diluted in blocking solution at 4 °C overnight. Cells were washed again three times with PBST (0.1% Tween) and incubated with 500 ng of DAPI and the secondary antibody (anti-mouse Alexa Fluor 488: 1:2,000) diluted in blocking solution for 1 h at room temperature in the dark. Cells were washed three times in PBST (0.1% Tween-20) before imaging.

### Flow cytometry analysis of eIF2α phosphorylation and p38 phosphorylation

Culture medium was exchanged with fresh medium 2 h before harvesting. Cells were collected by trypsinization, washed once with 1% BSA in PBS and fixed by slowly adding 3.7% formaldehyde in PBS and incubated for 10 min at room temperature. Cells were washed once with 1% BSA in PBS and incubated with ice-cold methanol for 10 min. Cells were washed once with 1% BSA in PBS and incubated with the primary antibody (eIF2α-p S51: 1:100, Abcam, ab32157; p38-p T180/Y182: 1:100, Cell Signaling, 9211) for 45 min at room temperature. Cells were washed twice with 1% BSA in PBS and incubated with the secondary antibody (anti-rabbit Alexa Fluor 633: 1:200, Thermo Fisher Scientific, A21070) for 45 min at room temperature. Cells were washed three times with 1% BSA in PBS and resuspended in PBS; fluorescence intensity was measured on an Attune NxT flow cytometer.

### Inhibitor treatments

Cells were treated with 1.25 µM GCN2i (A-92, Axon Medchem, Axon1386), 4 nM PERKi (GSK2606414, Merck, 516535), 1 µM p38i (SB203580, Cell Signaling, 5633) or 50 nM ISRIB (Sigma-Aldrich, SML0843). To induce p38 phosphorylation in positive control experiments, cells were treated with 0.05 mg L^−1^ ANS (Sigma-Aldrich, A9789) for 15 min before harvesting. To induce eIF2α phosphorylation, cells were treated with 2.5 µM tunicamycin (Sigma-Aldrich, T7765) for 2 h before harvesting.

### Polysome profiling and protein analysis of polysome fractions

Cells were grown in a 10-cm dish until ~80% confluency and cell medium was exchanged 2 h before harvesting. For ribosome runoff experiments, 2 µg ml^−1^ HAR (Cayman Chemical, 15361) was added to culture medium for 2.5 min or 2 h before harvesting. To induce ribosome collisions, 0.05 mg L^−1^ ANS (Sigma-Aldrich, A9789) was added to culture medium 15 min before harvesting. Cells were quickly washed with ice-cold PBS supplemented with 10 mM MgCl_2_ and 100 µg ml^−1^ CHX and snap-frozen in liquid nitrogen. Cells were harvested in lysis buffer containing 50 mM HEPES pH 7.4, 150 mM KCl, 15 mM MgCl_2_, 1% Triton X-100, 1 mM DTT, 1× protease and phosphatase inhibitors (10 µg ml^−1^ aprotinin, 20 µM leupeptin, 2.5 µM pepstatin A, 0.5 mM AEBSF and 1× phosphatase inhibitor cocktail (Cell Signaling, 5870)), 1 mM TCEP and 10 mM NEM. Cell extracts were directly loaded on 10–50% sucrose gradients in 50 mM HEPES pH 7.5, 150 mM KCl, 15 mM MgCl_2_, 1 mM DTT and 1 mM TCEP and 1.2-ml fractions were collected. Protein was extracted by TCA, acetone and ethanol precipitation. Then, 5% sodium deoxycholate was added to each fraction and incubated on ice for 30 min. Next, 20% TCA was added in a 1:1 ratio vortexed and incubated for another 30 min on ice. Protein pellets were collected by centrifugation for 30 min at 13,000*g* at 4 °C. Pellet was washed once with 100% acetone and dissolved in 4× Laemmli sample buffer. One quarter of each fraction was loaded per well.

### MS sample preparation

Inducible hiPS cells and inducible HEK293 cells were cultured as described above and 5 × 10^6^ cells were collected for liquid chromatography (LC)–MS/MS measurement in data-dependent acquisition (DDA) mode. For comparing protein levels in differentiated cells, inducible hiPS cells and NPCs were cultured in the presence of 2 µM doxycycline and differentiated into CMs and neurons, respectively, and 5 × 10^6^ cells were collected for LC–MS/MS measurement in data-independent acquisition (DIA) mode. Cell pellets were resuspended in 400 µl of SDC buffer (1% sodium deoxycholate, 40 mM 2-chloroacetamide, 10 mM TCEP and 100 mM Tris, pH 8.0) and incubated at 95 °C for 2 min followed by sonication in a Bioruptor plus system (Diagenode, UCD-300) ten times for 30 s each at high intensity. Heating and sonification were repeated; then, the samples were diluted with 200 µl of water (LC–MS grade) and supplemented with 1 µg of LysC (Wako Cat, 129-02541) followed by incubation at 37 °C for 2 h. After the addition of 2 µg of trypsin (Promega, V511A), the samples were digested overnight at 37 °C, acidified with trifluoroacetic acid (final concentration of 1%) and centrifuged to remove the precipitated SDC. The peptide mixture was either purified using SCX stage tips to yield desalted peptides or the peptides were fractionated into three fractions using SCX stage tips (Empore, 2251). For the fractionation by SCX stage tips, a buffer with increasing salt and pH were used (buffer 1: 150 mM ammonium acetate, 0.5% formic acid and 20% acetonitrile, pH 4.5; buffer 2: 300 mM ammonium acetate, 0.5% formic acid and 20% acetonitrile, pH 5.2; buffer 3: 5% ammonia and 80% acetonitrile) Desalted or fractionated peptides were vacuum-dried and analyzed by LC–MS/MS. Peptides were loaded onto a 30-cm column (inner diameter: 75 μm, packed in-house with ReproSil-Pur C18-AQ 1.9-μm beads; Dr. Maisch, r119.aq) using an Easy-nLC 1200 (Thermo Fisher Scientific) at 60 °C.

For DDA measurement, eluting peptides were directly sprayed onto the QExactive HF MS instrument (Thermo Fisher Scientific). Peptides were loaded in buffer A (0.1% formaldehyde) at 400 nl min^−1^ and the percentage of buffer B (80% acetonitrile and 0.1% formaldehyde) was increased from 2% to 30% over 120 min followed by an increase to 60% over 10 min and then 95% over the next 5 min. Finally, the percentage of buffer B was maintained for another 5 min. The MS instrument was operated in a DDA mode with survey scans from 300 to 1,650 *m*/*z* (resolution of 60,000 at *m*/*z* = 200) and up to ten of the top precursors were selected and fragmented using higher-energy collisional dissociation with a normalized collision energy of value of 28. The MS2 spectra were recorded at a resolution of 15,000 (at *m*/*z* = 200). The automatic gain control targets for MS1 and MS2 scans were set to 3 × 10^6^ and 1 × 10^5^, respectively, with maximum injection times of 100 and 60 ms for MS and MS2 scans, respectively.

For DIA measurement, eluting peptides were directly sprayed onto the timsTOF Pro (Bruker Daltonic). Peptides were loaded in buffer A at 400 nl min^−1^ and the percentage of buffer B was ramped from 5% to 25% over 90 min followed by a ramp to 35% over 30 min, a ramp to 58% over 5 min and a final ramp to 95% over the next 5 min, before maintaining at 95% for another 5 min. Data acquisition on the timsTOF Pro was performed using timsControl. The MS instrument was operated in DIA parallel accumulation serial fragmentation (PASEF) mode. Analysis was performed in a mass scan range from 100 to 1,700 *m*/*z* and an ion mobility range from 1/*K*_0_ = 0.47 to 1.60 V s cm^−2^ using equal ion accumulation and ramp time in the dual trapped ion mobility spectrometry (TIMS) analyzer of 100 ms each at a spectrum rate of 9.52 Hz. DIA PASEF scans were acquired a mass scan range from 350.2 to 1,199.9 Da and an ion mobility range from 1/*K*_0_ = 0.47 to 1.60 V s cm^−2^. Collision energy was ramped linearly as a function of the mobility from 45 eV at 1/*K*_0_ = 1.60 V s cm^−2^ to 27 eV at 1/*K*_0_ = 0.47 V s cm^−2^. In total, 42 DIA PASEF windows were distributed to one TIMS scan each at switching Th precursor isolation windows.

### RNA-seq library construction

First, 250 ng of total RNA was used for library preparation with the Universal Plus RNA-seq with NuQuant kit (Tecan, 0361). The fragment size of final libraries was determined on an Agilent TapeStation and the concentration was quantified using the Qubit dsDNA HS assay. Then, 80-bp single-end sequencing was performed on a NextSeq 500 platform (Illumina).

### Ribosome profiling library construction

Ribosome footprint libraries were prepared as previously described^50,121^ with minor modifications. Cells were grown in a 10-cm dish until ~80% confluency and the cell medium was exchanged 2 h before harvesting. Cells were quickly washed with ice-cold PBS supplemented with 10 mM MgCl_2_ and 100 µg ml^−1^ CHX and snap-frozen in liquid nitrogen. Cells were thawed on ice and scraped off the plate in 400 µl of polysome lysis buffer (20 mM Tris pH 7.4, 150 mM NaCl, 5 mM MgCl_2_, 1% Triton X-100, 1 mM DTT, 100 µg ml^−1^ CHX, 25 U per ml Turbo DNase (Thermo Fisher Scientific, AM2238) and 0.1% NP-40). Samples were vortexed vigorously, triturated through a 26G needle and spun down for 7 min at 16,000*g* at 4 °C. The supernatant was transferred to a new tube and RNA concentration was measured with the Qubit RNA HS kit. Aliquots of 20 µg of RNA in 200 µl of polysome lysis buffer were snap-frozen and stored at −80 °C. Then, 20 µg of RNA in 200 µl of polysome lysis buffer was digested with 50 U of RNase I (Thermo Fisher Scientific, AM2295) for 45 min at 2,000 rpm at 22 °C in a thermoblock. RNase I digestion was stopped by the addition of 100 U of Superase-In (Thermo Fisher Scientific, AM2694). Cell extracts were mixed by pipetting and 200 µl of extract was underlaid with 0.9 ml of a 1 M sucrose in polysome lysis buffer cushion, followed by centrifugation for 75 min at 650,000*g* at 4 °C in a S120-AT2 rotor (Thermo Fisher Scientific). The ribosomal pellet was dissolved in 400 µl of LiDS–LET lysis buffer and RNA was isolated by phenol extraction. Then, 3 µg of RNA was mixed with 2× RNA loading dye (45% formamide, 0.25× TBE and 0.025% SDS), incubated for 3 min at 90 °C and loaded on 15% polyacrylamide, 7 M urea and 1× TBE gels. Fragments in the range of 19–32 nt (monosome footprint libraries) and 50–80 nt (long footprint libraries) were excised from the gel on the basis of size markers and gel slices were crushed with a disposable pestle. RNA was eluted from gel slices with 400 µl of gel elution buffer (0.3 M sodium acetate pH 4.5, 0.25% SDS and 1 mM EDTA, pH 8.0) by incubation for 10 min at 65 °C, followed by snap-freezing on dry ice for 10 min, thawing for 5 min at 65 °C and an overnight elution at room temperature on a rotating wheel. Gel debris was removed by a Spin-X filter (Corning) and RNA was purified by ethanol precipitation. Size-selected RNA was dephosphorylated for 45 min at 37 °C using 5 U of T4 PNK (NEB, M0201S) and directly mixed with preadenylated adaptors containing five random nucleotides at their 5′ ends (Supplementary Table 5)^121^ in 1× T4 RNA ligase buffer (25% PEG-8000, 20 U of Superase-In and 200 U of T4 RNA ligase 2, truncated KQ (NEB, M0373S)). The linker ligation mix was incubated for 3 h at 25 °C and products were pooled and concentrated with the oligo clean and concentrator kit (Zymo Research, D4060). Ligation products were size-selected on a 12% polyacrylamide, 7 M urea and 1× TBE gel and purified as above in gel elution buffer. The sample concentration was quantified with the Nanodrop and 50 ng of the linker-ligated sample was used for rRNA depletion using the Ribo-seq riboPOOL h/m/r depletion kit (siTOOLs, dp-K024-000050).

For cDNA library generation, linker-ligated footprints were annealed with RT primer (Supplementary Table 5) at 65 °C for 5 min and reverse-transcribed for 30 min at 50 °C in 1× Protoscript II buffer containing 0.5 mM deoxynucleotide triphosphates, 10 mM DTT, 20 U of Superase-In and 200 U of Protoscript II (NEB, E6560S). The RNA was hydrolyzed by addition of 0.1 M NaOH and incubation for 5 min at 90 °C and cDNA products were size-selected on a 12% polyacrylamide, 7 M urea and 1× TBE gel. The DNA was eluted from crushed gel slices for 60 min at 1,500 rpm at 70 °C in 1× TE buffer. The crushed gel was removed by filtering samples through a Spin-X filter (Corning) and cDNA was purified by ethanol precipitation.

The cDNA was circularized by addition of 3 µM recombinant TS2126 RNA ligase 1 (commercially sold as CircLigase)^122^ in circularization buffer (50 µM ATP, 2.5 mM MnCl_2_, 50 mM MOPS pH 7.5, 10 mM KCl, 5 mM MgCl_2_, 1 mM DTT and 1 mM betaine) and incubation for 3 h at 60 °C, followed by heat inactivation for 10 min at 80 °C. Libraries were amplified from circularized cDNA using a common forward primer, an index-containing reverse primer (Supplementary Table 5) and KAPA HiFi DNA polymerase (Roche) in 1× HiFi buffer. Samples were initially denatured at 95 °C for 3 min, followed by 12 cycles of 98 °C for 20 s, 62 °C for 30 s and 72 °C for 15 s at a ramp rate of 3 °C s^−1^. PCR products were size-selected on an 8% polyacrylamide and 1× TBE gel. The DNA was eluted from crushed gel slices on a rotating wheel overnight at room temperature in DNA elution buffer (300 mM NaCl, 10 mM Tris-Cl pH 7.5 and 0.2% Triton X-100). Gel debris was removed by filtering samples through a Spin-X filter (Corning) and DNA was purified by ethanol precipitation. Libraries were quantified with the Qubit dsDNA HS kit and 75–86-bp single-end sequencing was performed on a NextSeq 500 or a NovaSeq 6000 platform (Illumina).

### CRISPRi screen data analysis

sgRNA-seq datasets were processed following the ScreenProcessing pipeline (https://github.com/mhorlbeck/ScreenProcessing)^31^. For primer set A, the script was run with ‘--trim_start 1 --trim_end 20’ to select the first 20 nt; for primer set B, ‘--trim_start 20 --trim_end 39’ was used to select the last 20 nt. We modified the fastgz_to_counts.py script by adding a parameter ‘--revcomp’, which we additionally supplied to the counting for primer set B to allow the alignment of the reverse complement of these sequences originating from library construction with a reverse primer. Following sgRNA counting, process_experiments.py was run for phenotype calculations. sgRNA analysis was conducted for each cell and screen type by comparing matched doxycycline-induced samples to uninduced (no doxycycline) control samples cultured in parallel for the duration of the experiment. Pseudocounts of one were added for zero-count sgRNAs only using ‘pseudocount_behavior = zeros only’ and ‘pseudocount = 1’. sgRNA-level data were collapsed by transcript using ‘collapse_to_transcripts = true’. sgRNAs that had fewer than 50 reads were excluded from the analysis by setting ‘minimum_reads = 50’. Gene-avegared sgRNA log_2_ fold changes were calculated by normalizing sgRNA log_2_ enrichment of the average top three sgRNAs using ‘calculate_ave = true’ and ‘best_n = 3’. The Mann–Whitney *P* value was calculated using the average log_2_ fold change from all nine sgRNAs targeting the same gene TSS compared to nontargeting controls by setting ‘calculate_mw = true’. For defining gene essentiality, gene-averaged log_2_ fold changes of genes with two alternative TSSs were collapsed by choosing the stronger magnitude effect with a Mann–Whitney *P* value ≤ 0.1. For comparison of shared essential genes, datasets from CRISPR-inferred common essential genes in DepMap 23Q4 and genome-wide CRISPRi screens in WTC11 hiPS cells^35^ and in WTC11 hiPS cell-derived neurons^36^ (false discovery rate (FDR) ≤ 0.1) were subset for the 262 genes targeted in our screens and the overlap was visualized with UpSetR version 1.4.0 in R version 4.2.2.

### MS data analysis

DDA raw data were processed using the MaxQuant computational platform (version 2.2.0.0) with standard settings applied for Orbitrap data. DIA raw data were processed using Spectronaut 18.0 in directDIA+ (library-free) mode. Briefly, the peak list was searched against a predicted library of the human database from UniProt (downloaded in 2023). Cysteine carbamidomethylation was set as static modification and methionine oxidation and N-terminal acetylation were set as variable modifications. The match-between-run option was enabled and proteins were quantified across samples using label-free quantification (MaxLFQ) at the MS2 level. The data were further processed using Perseus version 2.0.10.0 including the Proteomic Ruler plugin (https://pubmed.ncbi.nlm.nih.gov/25225357/). Protein copy numbers were estimated using the default settings of the Proteomic Ruler plugin. Contaminants, reverse proteins and proteins only identified by site or by only one peptide were filtered out. Replicates were annotated to specific cell types. LFQ or copy numbers were log_2_-transformed and filtered for values in two replicates in at least one cell type. Data were imputed with default settings. The log_2_ fold changes and *P* values were calculated using a two-sided *t*-test with default settings and an FDR = 0.01.

### RNA-seq data analysis

Potential 3′ adaptors in RNA-seq reads were removed using TrimGalore version 0.6.4 with default settings, retaining reads with length ≥ 20 nt. Alignment of trimmed reads to the GRCh38 genome was performed with STAR version 2.6.1c to an index built including the GENCODE comprehensive annotation GTF file to guide splice-aware alignment. The following parameters were used for alignment with STAR to ensure no more than one mismatch per read and retention of only uniquely mapped reads: ‘--outSAMtype BAM SortedByCoordinate --outFilterMultimapNmax 1 --outFilterMismatchNmax 1’. Read counts per gene were then generated using featureCounts version 1.6.2 and only the protein-coding gene annotation subset from GENCODE. This count table was then used as input for DESeq2 version 1.38.1 to perform differential gene expression analysis. DESeq2 was run with default settings to quantify differential expression between conditions. Using the resulting log_2_ fold changes, heatmaps comparing gene differential expression between conditions were assembled using ComplexHeatmap version 2.14.0 (ref. ^123^), where only significant values were retained (adjusted *P* ≤ 0.05). GO enrichment was analyzed for significantly altered log_2_ fold changes (adjusted *P* ≤ 0.05) using clusterProfiler version 4.4.4 (ref. ^124^) with a significance cutoff of ≤0.01 (Benjamini–Hochberg method).

### Ribosome profiling data analysis

A custom reference transcriptome annotation was built for the GRCh38.p13 genome on the basis of MANE and the best-scoring transcript annotated in APPRIS (https://apprisws.bioinfo.cnio.es/pub/current_release/datafiles/homo_sapiens/GRCh38/appris_data.appris.txt)^125^. For genes without a MANE-annotated transcript, the best-scoring transcript in APPRIS was chosen as the principal isoform. For multiple best-scoring transcripts associated with the same gene, the longest isoform was selected. When multiple best-scoring transcripts had the same length, a random one was chosen as the principal isoform. This reference set was further filtered for transcripts with coding regions ending with a UAG, UAA or UGA stop codon, having a three-base periodicity and not containing unidentified bases in the open reading frame. Known transcripts with non-AUG start codons (for example, EIF4G2) or containing UGA codons recoded as selenocysteine were retained and the remaining set was curated by manually removing misannotations that resulted in multiple transcripts per Ensembl gene identifier and filtering out 18 transcripts associated with pseudoautosomal regions on the Y chromosome. The resulting reference contained 19,759 protein-coding transcripts and was used for ribosome footprint and matched RNA-seq read alignment.

Sequencing libraries were demultiplexed and adaptor-trimmed with Cutadapt version 2.5 with the following command: ‘--no-indels -q 30,30 --trimmed-only’. Reads were further trimmed to remove the two 5′-RN nucleotides introduced by circularization from the RT primer with ‘-u 2’. Trimmed reads longer than 10 nt were aligned to a human ribosomal RNA reference using bowtie version 1.2.2 (ref. ^126^) with ‘-p 40 -S --best’. rRNA-filtered reads were aligned to GRCh38.p13 using STAR version 2.6.1c (ref. ^127^) with the following options: ‘--outFilterMultimapNmax 1 --outSAMtype BAM SortedByCoordinate --outFilterMismatchNmax 0 --alignEndsType Local --seedSearchStartLmax 14 --alignIntronMax 10000 --outFilterIntronMotifs RemoveNoncanonicalUnannotated --quantMode TranscriptomeSAM --outSAMattributes NH HI AS nM NM MD’. Aligned read files were sorted and indexed with SAMtools version 1.11.

The A-site codon in each mapped read was identified with Scikit-ribo^30,128^. Kallisto 0.44.0 with parameters ‘-b 100 --single -l 180 -s 20 -t 40’ was used to quantify transcript abundances in transcripts per million (TPM) from RNA-seq data on the basis of MANE and APPRIS annotation. To make the GRCh38 GTF compatible with Scikit-ribo, transcript and UTR annotations were removed. For each transcript, the start codon in the first exon and the stop codon in the last exon were adjusted to represent transcript start and end coordinates, taking into account the gene strand. For this, only transcripts with a single annotated start codon and a single annotated stop codon in the hg38 GTF were retained, thus excluding 60 transcripts from the reference transcriptome. For monosome footprint libraries, read lengths corresponding to ribosomes with vacant (20–22 nt) or occupied (29–32 nt) ribosomal A sites were included in the subsequent analysis (‘-s 20 -l 22’ and ‘-s 29 -l 32’).

### Ribosomal pause site analysis

After A-site identification in monosome footprint libraries, the average footprint coverage for each transcript was calculated by summing the number of 20–22-nt and 29–32-nt monosome footprints mapped to the CDS and dividing this sum by the CDS length in codons. Only transcripts with an average coverage of ≥0.5 reads per codon in each replicate in the two conditions compared were included in subsequent analyses. To identify ribosomal pauses, reads at each codon position were averaged between replicates and rounded to the nearest integer. Then, the codon coverage per transcript in the average profile and in both replicates in each condition was transformed into *Z* scores using Python version 3.7. Codons with a *Z* score ≥ 5.0 in the average profile were considered as ribosomal pause sites^83^. To further increase the confidence of pause site identification, only pauses detected in the average profile and in both biological replicates at the same codon position or at one of the two flanking codons were retained. Differential ribosome pauses were then identified as previously described^129^. Briefly, the average profile generated from biological replicates in each condition was used to calculate a pause score for each codon position by dividing the number of footprint counts in that position for the average ribosome footprint count along the CDS (excluding the first five and last five codons because of effects on ribosome occupancy related to initiation and termination). A 2 × 2 contingency table was then used to perform a Fisher’s exact test and compare the ratio of reads in mutant or treated and control samples at each codon position to the ratio of mapped reads at all other positions in the transcript by calculating an odds ratio and an adjusted *P* value with a Benjamini–Hochberg correction for multiple hypothesis testing. Differential pause sites were defined as codon positions with a *Z* score ≥ 5 in the mutant, an odds ratio ≠ 1 and an adjusted *P* value < 0.05. Increased differential pauses were defined as those codon positions having a *Z* score ≥ 5 in the mutant, an odds ratio > 1 and a pause score in the mutant or treated sample greater than in the control. Sequence motifs in differential pause sites were analyzed with ggseqlogo version 0.1 (ref. ^130^). Amino acid sequence logos for pauses in the first five and in the last five codons of CDSs were generated by extracting random amino acids with a probability of 1/20.

Long footprint reads preprocessed and aligned as monosome footprints were analyzed with riboWaltz version 1.2.0. Heatmaps of read 5′ ends around start and stop codons stratified by length were generated using rends_heat. P-site prediction of reads with length between 40 and 70 nt was performed using psite, specifying ‘start = false’ so that the second-to-last codon was used for the prediction instead of the default start codon. Length-filtered reads were updated with these P-site predictions using psite_info. Metagene profiles of P-site coverage from disome footprints were plotted using metaprofile_psite with frequency-normalized P-site counts by specifying ‘frequency = true’.

### Reporting summary

Further information on research design is available in the Nature Portfolio Reporting Summary linked to this article.

## Online content

Any methods, additional references, Nature Portfolio reporting summaries, source data, extended data, supplementary information, acknowledgements, peer review information; details of author contributions and competing interests; and statements of data and code availability are available at 10.1038/s41594-025-01616-3.

## Supplementary information

Reporting Summary

Supplementary Tables Supplementary Table 1: CRISPRi screen analysis results per gene and sgRNA. Supplementary Table 2: Differential expression analysis at mRNA transcript level for inducible hiPS cell and inducible HEK293 cell knockdown lines. Supplementary Table 3: Differential expression analysis at mRNA transcript level for ZNF598^RING^-expressing inducible hiPS cells. Supplementary Table 4: Differential pause site analysis for monosome footprint libraries of ZNF598^RING^-expressing and *ZNF598*-knockdown inducible hiPS cells. Supplementary Table 5: Primer sequences for qPCR and library construction. Supplementary Table 6: sgRNA sequences for individual gene knockdown experiments.

## Source data

Source Data Fig. 1 Statistical source data.

Source Data Fig. 2 Statistical source data.

Source Data Fig. 3 Statistical source data.

Source Data Fig. 4 Statistical source data.

Source Data Fig. 4 Uncropped western blots.

Source Data Fig. 5 Statistical source data.

Source Data Fig. 5 Uncropped western blots.

Source Data Fig. 6 Statistical source data.

Source Data Fig. 6 Uncropped western blots.

Source Data Extended Data Fig. 1 Statistical source data.

Source Data Extended Data Fig. 1 Uncropped western blots.

Source Data Extended Data Fig. 2 Statistical source data.

Source Data Extended Data Fig. 2 Uncropped western blots.

Source Data Extended Data Fig. 3 Statistical source data.

Source Data Extended Data Fig. 4 Statistical source data.

Source Data Extended Data Fig. 5 Statistical source data.

Source Data Extended Data Fig. 6 Statistical source data.

Source Data Extended Data Fig. 6 Uncropped western blots.

Source Data Extended Data Fig. 7 Statistical source data.

Source Data Extended Data Fig. 8 Statistical source data.

Source Data Extended Data Fig. 8 Uncropped western blots.

## Data Availability

High-throughput sequencing data were deposited to the Gene Expression Omnibus under accession number GSE246419. MS data were deposited to the ProteomeXchange Consortium through the PRIDE partner repository under accession code PXD044928. The GRC38.p13 human genome assembly is available from https://www.ncbi.nlm.nih.gov/datasets/genome/GCF_000001405.39/. Source data are provided with this paper.

## Code availability

Customized Scikit-ribo version 0.2.4bl for use on the human genome is available from GitHub (https://github.com/nedialkova-lab/scikit-ribo-ext).
