# Supplementary material for: Comparative CRISPRi screens reveal a human stem cell dependence on mRNA translation-coupled quality control
Source: Nat Struct Mol Biol. 2025 Jul 11;32(10):1932–46. doi: 10.1038/s41594-025-01616-3 (PMC12527931; doi:10.1038/s41594-025-01616-3)
Supplement: Supplementary file 1 — Reporting Summary [file 41594_2025_1616_MOESM1_ESM.pdf]

nature portfolio

Corresponding author(s): Danny D. Nedialkova

Last updated by author(s): May 1, 2025

Reporting Summary

Nature Portfolio wishes to improve the reproducibility of the work that we publish. This form provides structure for consistency and transparency in reporting. For further information on Nature Portfolio policies, see our Editorial Policies and the Editorial Policy Checklist.

Statistics

For all statistical analyses, confirm that the following items are present in the figure legend, table legend, main text, or Methods section.

n/a	Confirmed	
☐	☒	The exact sample size (*n*) for each experimental group/condition, given as a discrete number and unit of measurement
☐	☒	A statement on whether measurements were taken from distinct samples or whether the same sample was measured repeatedly
☐	☒	The statistical test(s) used AND whether they are one- or two-sided<br>*Only common tests should be described solely by name; describe more complex techniques in the Methods section.*
☐	☒	A description of all covariates tested
☐	☒	A description of any assumptions or corrections, such as tests of normality and adjustment for multiple comparisons
☐	☒	A full description of the statistical parameters including central tendency (e.g. means) or other basic estimates (e.g. regression coefficient) AND variation (e.g. standard deviation) or associated estimates of uncertainty (e.g. confidence intervals)
☐	☒	For null hypothesis testing, the test statistic (e.g. *F*, *t*, *r*) with confidence intervals, effect sizes, degrees of freedom and *P* value noted<br>*Give P values as exact values whenever suitable.*
☒	☐	For Bayesian analysis, information on the choice of priors and Markov chain Monte Carlo settings
☒	☐	For hierarchical and complex designs, identification of the appropriate level for tests and full reporting of outcomes
☐	☒	Estimates of effect sizes (e.g. Cohen's *d*, Pearson's *r*), indicating how they were calculated

Our web collection on statistics for biologists contains articles on many of the points above.

Software and code

Policy information about availability of computer code

Data collection	Western blot images were collected with iBright Analysis Software v1.8.2 (Thermo Fisher).

Data analysis	R v4.2.2<br>Python v3.7<br>Customized scikit-ribo v0.2.4b1 for use on human genome (https://github.com/nedialkova-lab/scikit-ribo-ext).<br>STAR v2.6.1.c<br>bowtie v1.2.2<br>kallisto v0.44.0<br>featureCounts v1.6.2<br>riboWaltz v1.2.0<br>cutadapt v2.5<br>TrimGalore v0.6.4<br>samtools v1.11<br>DESEq2 v1.38.1<br>ComplexHeatmap v2.14.0<br>clusterProfiler v4.4.4<br>UpSetR v1.4.0<br>ggseqlogo v0.1<br>Flowjo v10.8

For manuscripts utilizing custom algorithms or software that are central to the research but not yet described in published literature, software must be made available to editors and reviewers. We strongly encourage code deposition in a community repository (e.g. GitHub). See the Nature Portfolio guidelines for submitting code & software for further information.

Data

Policy information about availability of data

All manuscripts must include a data availability statement. This statement should provide the following information, where applicable:

- Accession codes, unique identifiers, or web links for publicly available datasets
- A description of any restrictions on data availability
- For clinical datasets or third party data, please ensure that the statement adheres to our policy

High-throughput sequencing data has been deposited in the Gene Expression Omnibus Database (GSE246419). Mass Spectrometry data has been deposited to the ProteomeXchange Consortium via the PRIDE partner repository (PXD044928). The GRC38.p13 human genome assembly is available at https://www.ncbi.nlm.nih.gov/datasets/genome/GCF_000001405.39/.

Research involving human participants, their data, or biological material

Policy information about studies with human participants or human data. See also policy information about sex, gender (identity/presentation), and sexual orientation and race, ethnicity and racism.

Reporting on sex and gender	N/A
Reporting on race, ethnicity, or other socially relevant groupings	N/A
Population characteristics	N/A
Recruitment	N/A
Ethics oversight	N/A

Note that full information on the approval of the study protocol must also be provided in the manuscript.

Field-specific reporting

Please select the one below that is the best fit for your research. If you are not sure, read the appropriate sections before making your selection.

☒ Life sciences ☐ Behavioural & social sciences ☐ Ecological, evolutionary & environmental sciences

For a reference copy of the document with all sections, see nature.com/documents/nr-reporting-summary-flat.pdf

Life sciences study design

All studies must disclose on these points even when the disclosure is negative.

Sample size	No statistical method was used to determine appropriate sample sizes. Sample size selection was guided by standard practices in the field and own experience. Sample sizes are specified in the related figure legends.

Data exclusions	No data was excluded.
Replication	The number of experimental replicates are indicated in the corresponding figure legends. All attempts of replications were successful.
Randomization	No randomization was performed. This study was carried out in the kucg_2 hiPSC line and its differentiated counterparts, as well as in HEK293. Covariates control is not applicable due to the small number of cell lines used.
Blinding	Measurements were conducted using automated equipment, such as gel scanners, sequencers, and mass spectrometers, eliminating the need for blinding. Investigators were not blinded during data collection or data analysis as the pronounced differences in cell morphology and cellular responses to gene knockdown clearly revealed sample identities, rendering blinding impractical.

Reporting for specific materials, systems and methods

We require information from authors about some types of materials, experimental systems and methods used in many studies. Here, indicate whether each material, system or method listed is relevant to your study. If you are not sure if a list item applies to your research, read the appropriate section before selecting a response.

Materials & experimental systems

n/a	Involved in the study
☐	☒ Antibodies
☐	☒ Eukaryotic cell lines
☒	☐ Palaeontology and archaeology
☒	☐ Animals and other organisms
☒	☐ Clinical data
☒	☐ Dual use research of concern
☒	☐ Plants

Methods

n/a	Involved in the study
☒	☐ ChIP-seq
☐	☒ Flow cytometry
☒	☐ MRI-based neuroimaging

Antibodies

Antibodies used

Anti-POU5F1 C-10 (1:400; Santa Cruz, #sc-5279)
Anti-NANOG P1-2D8 (1:200, Millipore, #MABD24)
Anti-PAX6 (1:200; Abcam #ab5790)
Anti-Nestin (1:200; R&D Systems, #MAB1259)
Anti-MAP2 (1:1000; Abcam, #ab92434)
Anti-CHAT (1:200; Abcam, #ab6168)
Anti-cTNT (1:5; CT3, deposited to the DSHB by Lin, J.J-C.)
Anti-ACTN2 (1:800; Sigma-Aldrich #A7811)
Anti-ZNF598 (1:1000, #ab135921 Abcam)
Anti-PELO F-4 (1:1000, #sc-393418 Santa Cruz Biotechnology)
Anti-HBS1L (1:1000, #HPA029729 Atlas Antibodies)
Anti-ASCC3 (1:1000, #A304-015A Bethyl Laboratories)
Anti-eIF2α (1:1000, #9722 Cell Signaling)
Anti-eIF2α-p S51 (1:1000, #ab32157 Abcam)
Anti-p38 (1:1000, #9212 Cell Signaling)
Anti-p38-p Thr180/Tyr182 (1:1000, #9211 Cell Signaling)
Anti-uS10 (1:1000, #ab151550 Abcam)
Anti-eS10 (1:1000, #ab133776, Abcam)
Anti-uS5 (1:1000, #A303-794A Bethyl Laboratories)
Anti-uS3 (1:1000, #A303-840A Bethyl Laboratories)
Goat anti-mouse Alexa Fluor 488 (1:2000; Thermo Fisher Scientific, #A-11001)
Goat anti-rabbit Alexa Fluor 488 (1:2000; Thermo Fisher Scientific, #A-11034)
Goat anti-mouse Alexa Fluor 633 (1:500; Thermo Fisher Scientific, #A-21052)
Anti-mouse IgG-HRP, 1:4000; Dianova, #115-035-003
Anti-rabbit IgG-HRP (1:4000; Dianova, #111-035-003)

Validation

Anti-POU5F1 C-10 (Santa Cruz, #sc-5279): negative staining of cells that are not expressing the marker in Gao, Behrens et al., Nature Cell Biology 2024 .

Anti-P1-2D8 (1:200, Millipore, #MABD24): according to the manufacturer, this monoclonal antibody is suitable for detecting human NANOG by immunohistochemistry; cited in 40 publications (https://www.sigmaaldrich.com/GB/en/product/mm/mabd24).

Anti-PAX6 (Abcam #ab5790): negative staining of cells that are not expressing the marker in Gao, Behrens et al., Nature Cell Biology 2024.

Anti-Nestin (R&D Systems, #MAB1259): negative staining of cells that are not expressing the marker in Gao, Behrens et al., Nature

Cell Biology 2024.

Anti-MAP2 (Abcam, #ab92434): negative staining of cells that are not expressing the marker in Gao, Behrens et al., Nature Cell Biology 2024.

Anti-CHAT (Abcam, #ab6168): negative staining of cells that are not expressing the marker in Gao, Behrens et al., Nature Cell Biology 2024.

Anti-cTNT (CT3, deposited to the DSHB by Lin, J.J-C.): negative staining of cells that are not expressing the marker in Gao, Behrens et al., Nature Cell Biology 2024.

Anti-ACTN2 (Sigma-Aldrich #A7811): negative staining of cells that are not expressing the marker in Gao, Behrens et al., Nature Cell Biology 2024.

Anti-ZNF598 (1:1000, #ab135921 Abcam): according to the manufacturer, this polyclonal antibody is suitable for detecting human ZNF598 by immunoblotting; cited in 3 publications (https://www.abcam.com/products/primary-antibodies/znf598-antibody-ab135921.html). Validated by lack of signal in samples in which ZNF598 expression was knocked down.

Anti-PELO F-4 (1:1000, #sc-393418 Santa Cruz Biotechnology): according to the manufacturer, this monoclonal antibody is suitable for detecting human PELO by immunoblotting; cited in 5 publications (https://www.scbt.com/de/p/pelo-antibody-f-4). Validated by lack of signal in samples in which PELO expression was knocked down.

Anti-HBS1L (1:1000, #HPA029729 Atlas Antibodies): according to the manufacturer, this polyclonal antibody is suitable for detecting human HBS1L by immunoblotting; cited in 10 publications (https://www.sigmaaldrich.com/DE/de/product/sigma/hpa029729).Validated by lack of signal in samples in which HBS1L expression was knocked down.

Anti-ASCC3 (1:1000, #A304-015A Bethyl Laboratories): according to the manufacturer, this polyclonal antibody is suitable for detecting human ASCC3 by immunoblotting; cited in 5 publications (https://www.biomol.com/de/produkte/antikoerper/primaerantikoerper/allgemein/anti-ascc3-a304-015a-t?number=A304-015A). Validated by lack of signal in samples in which ASCC3 expression was knocked down.

Anti-eIF2α (1:1000, #9722 Cell Signaling): according to the manufacturer, this polyclonal antibody is suitable for detecting human eIF2α by immunoblotting; cited in >600 publications (https://www.cellsignal.com/products/primary-antibodies/eif2a-antibody/9722).

Anti-eIF2α-p S51 (1:1000, #ab32157 Abcam): according to the manufacturer, this monoclonal antibody is suitable for detecting human phosphorylated eIF2α by immunoblotting; cited in >83 publications (https://www.abcam.com/en-de/products/primary-antibodies/eif2s1-phospho-s51-antibody-e90-ab32157#).

Anti-p38 (1:1000, #9212 Cell Signaling): according to the manufacturer, this polyclonal antibody is suitable for detecting human p38 by immunoblotting; cited in >2700 publications (https://www.cellsignal.com/products/primary-antibodies/p38-mapk-antibody/9212).

Anti-p38-p Thr180/Tyr182 (1:1000, #9211 Cell Signaling): according to the manufacturer, this polyclonal antibody is suitable for detecting human phosphorylated p38 by immunoblotting; cited in >1600 publications (https://www.cellsignal.com/products/primary-antibodies/phospho-p38-mapk-thr180-tyr182-antibody/9211).

Anti-RPS10 (= eS10, 1:1000, #ab151550 Abcam): according to the manufacturer, this monoclonal antibody is suitable for detecting human α-RPS20 (uS10) by immunoblotting; cited in 13 publications (https://www.abcam.com/products/primary-antibodies/rps10-antibody-epr8545-ab151550.html).

Anti-RPS20 (=uS10, 1:1000, #ab133776, Abcam): according to the manufacturer, this monoclonal antibody is suitable for detecting human α-RPS20 (uS10) by immunoblotting; cited in 18 publications (https://www.abcam.com/products/primary-antibodies/rps20-antibody-epr8716-ab133776.html).

Anti-RPS2 (=uS5, 1:1000, #A303-794A Bethyl Laboratories): according to the manufacturer, this polyclonal antibody is suitable for detecting human α-RPS2 (uS5) by immunoblotting; cited in 10 publications (https://www.biomol.com/de/produkte/antikoerper/primaerantikoerper/allgemein/anti-rps2-a303-794a-t?number=A303-794A).

Anti-RPS3 (=uS3, 1:1000, #A303-840A Bethyl Laboratories): according to the manufacturer, this polyclonal antibody is suitable for detecting human α-RPS3 (uS3) by immunoblotting; cited in 8 publications (https://www.biomol.com/de/produkte/antikoerper/primaerantikoerper/allgemein/anti-rps3-a303-840a-t?number=A303-840A).

Eukaryotic cell lines

Policy information about cell lines and Sex and Gender in Research

Cell line source(s)	The hiPSC HPSI0214i-kucg_2 (male) cell line was sourced from the HipSci Consortium (https://www.hipsci.org/) through the European Collection of Authenticated Cell Cultures (ECACC). HEK293 cells were a gift from Martin Dichgans (Institute for Stroke and Dementia Research, University Hospital, LMU Munich). Lenti-X™ 293T cells were obtained from Takara Bio (#632180).
Authentication	hiPSC, NPC, neurons, and cardiomyocytes were authenticated by the analysis of marker gene expression by fluorescence microscopy. HEK293 and Lenti-X™ 293T cells were not authenticated.

Mycoplasma contamination | All cell lines used in this study were tested negative for mycoplasma contamination.

Commonly misidentified lines (See ICLAC register) | No commonly misidentified lines were used.

Flow Cytometry

Plots

Confirm that:

☒ The axis labels state the marker and fluorochrome used (e.g. CD4-FITC).

☒ The axis scales are clearly visible. Include numbers along axes only for bottom left plot of group (a 'group' is an analysis of identical markers).

☒ All plots are contour plots with outliers or pseudocolor plots.

☒ A numerical value for number of cells or percentage (with statistics) is provided.

Methodology

Sample preparation

For growth assays, cells were trypsinized, resuspended in fresh medium and analyzed for GFP expression compared to a control without GFP expression. For stalling reporter assays, cells were trypsinized, resuspended in fresh medium and analyzed for GFP, mCherry, mOrange, and BFP expression compared to controls without fluorescent protein expression. For global protein synthesis measurements by ,HPG or OPP incorporation into nascent chains, cells were trypsinized, washed once with PBS, fixed in 3.7% formaldehyde in TBS, washed with TBS, and permeabilized for 15 minutes in TBS-0.5% Tween 20. HPG and OPP were labeled with click chemistry by a 30-minutes incubation in 100 mM Tris pH=8, 1 mM $CuSO_4$, 20 µM AF647-Picolyl-Azide, #CLK-1300 (Jena Bioscience), 100 mM ascorbic acid). Cells were washed three times in TBS-0.2% Tween 20 and fluorescence signal was analysed on an Attune Nxt flow cytometer. For DNA staining, cells were labeled using the EdU Flow Cytometry kit (Thermo Fisher Scientific) according to the manufacturer's instructions and fluorescence intensity was measured on an Attune Nxt flow cytometer. For eIF2α phosphorylation and p38 phosphorylation analyses, cells were trypsinized, washed once with 1 % BSA/PBS, fixed in 3.7% formaldehyde in PBS, washed with 1 % BSA in PBS and permeabilized for 10 minutes in 100% ice-cold methanol. Cells were washed once with 1% BSA in PBS and incubated with the primary antibody (eIF2α-p S51, 1:100, # ab32157 Abcam; p38-p Thr180/Tyr182, 1:100, #9211 Cell Signaling) for 45 minutes at room temperature. Cells were washed twice with 1% BSA in PBS and incubated with the secondary antibody (anti-rabbit Alexa Fluor 633, 1:200, Thermo Fisher Scientific, #A21070) for 45 minutes at room temperature. Cells were washed three times with 1% BSA/ PBS, resuspended in PBS and fluorescence intensity was measured on an Attune NxT flow cytometer.

Instrument | Attune NxT system

Software | FlowJo v10.8

Cell population abundance | We aquired > 10,000 cells in the FSC-A/ SSC-A gate using the Attune NxT system settings (removed cell debris, only one cell population per measurement).

Gating strategy

For all assays, cells were initially acquired with FSC-A/ SSC-A setting to remove cell debris (only one cell population per measurement). Single cells were selected with FSC-H/FSC-A gates.

For growth assays, the percentage of GFP-positive cells (BL1, Attune NxT) were then quantified compared to a control without GFP expression.

For stalling reporter assays, the cells were then subsetted for cells only expressing mCherry (YL2, Attune NxT) and GFP (BL1, Attune NxT) compared to cells not expressing any fuorescent protein. The ratio of mOrange:BFP (YL1 and VL1, Attune NxT) was then either plotted per condition, or the median fluorescent intensity was quantified using FlowJo v10.8 and changes were quantified relative to cells expressing a non-targeting sgRNA as control. For compensation of different fluorophores, we used cells expressing only one of each fluorophore under the same promoter. Compensation was automatically applied on the Attune NxT system immediately before measuring the probes.

For global protein synthesis measurement, the A647 content (RL1, Attune NxT) was quantified compared to a control without A647 expression. The median fluorescent intensity was quantified using FlowJo v10.8.

For eIF2α phosphorylation and p38 phosphorylation analyses the A647 content (RL1, Attune NxT) was quantified compared to control cells without any knockdown or construct expression. The median fluorescent intensity was quantified using FlowJo v10.8.

☒ Tick this box to confirm that a figure exemplifying the gating strategy is provided in the Supplementary Information.
