# Supplementary material for: Comparative CRISPRi screens reveal a human stem cell dependence on mRNA translation-coupled quality control
Source: Nat Struct Mol Biol. 2025 Jul 11;32(10):1932–46. doi: 10.1038/s41594-025-01616-3 (PMC12527931; doi:10.1038/s41594-025-01616-3)

Source Data Fig. 4g

Ponceau (total protein)

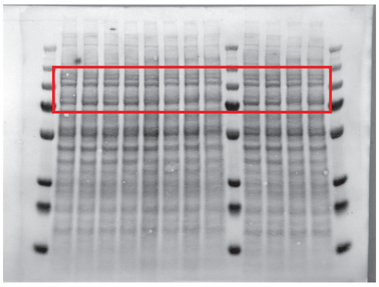

Ponceau (total protein)

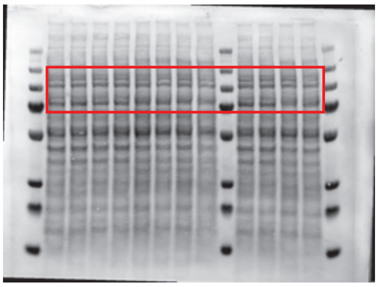

anti-eIF2α-p

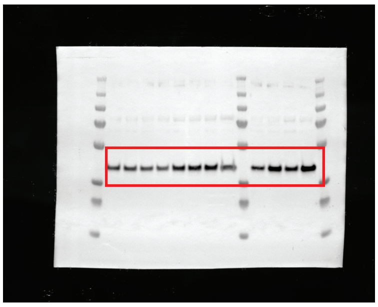

anti-p38-p

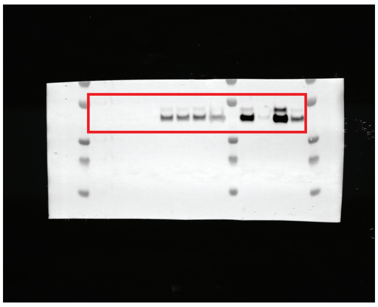

anti-eIF2α

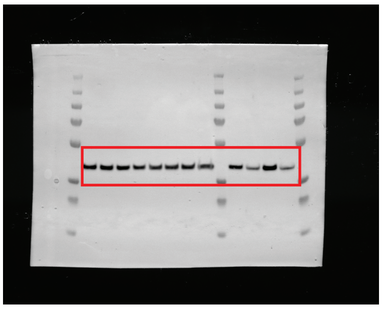

anti-p38

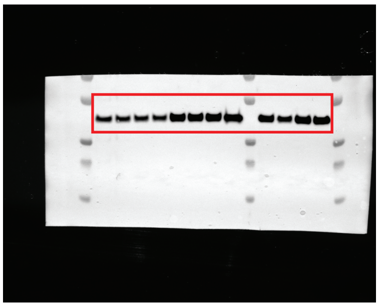

Supplement: Supplementary file 7 — Uncropped western blots. [file 41594_2025_1616_MOESM7_ESM.pdf]
