# Supplementary figures and images for: Comparative CRISPRi screens reveal a human stem cell dependence on mRNA translation-coupled quality control
Source: Nat Struct Mol Biol. 2025 Jul 11;32(10):1932–46. doi: 10.1038/s41594-025-01616-3 (PMC12527931; doi:10.1038/s41594-025-01616-3)

Source Data Fig. 5b

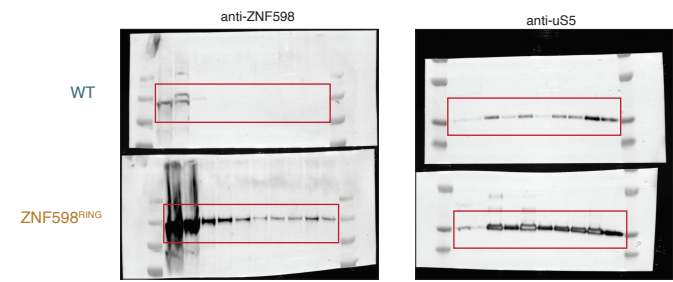

Supplement: Supplementary file 9 — Uncropped western blots. [file 41594_2025_1616_MOESM9_ESM.pdf]

anti-eS10

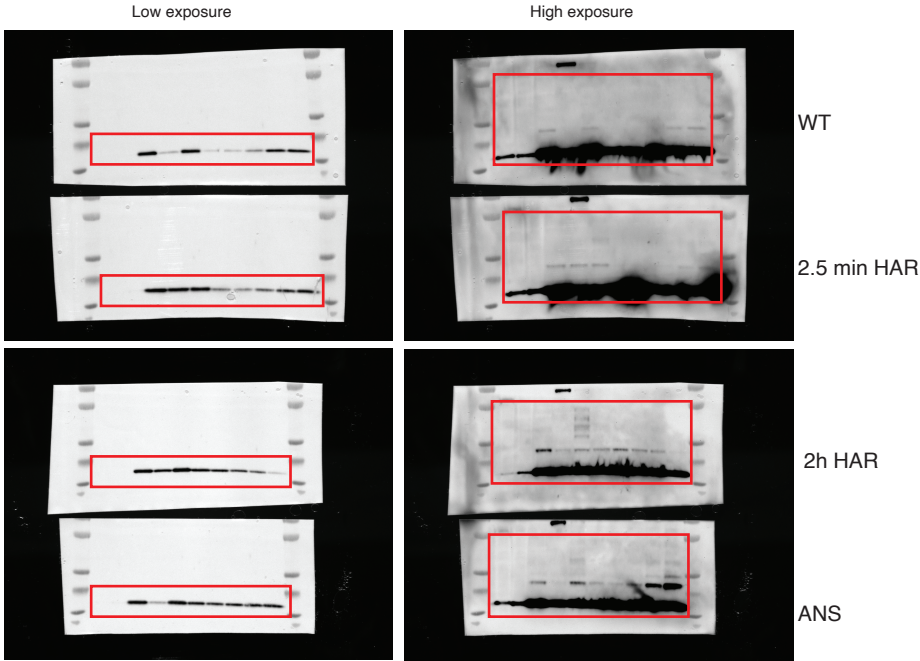

anti-uS10

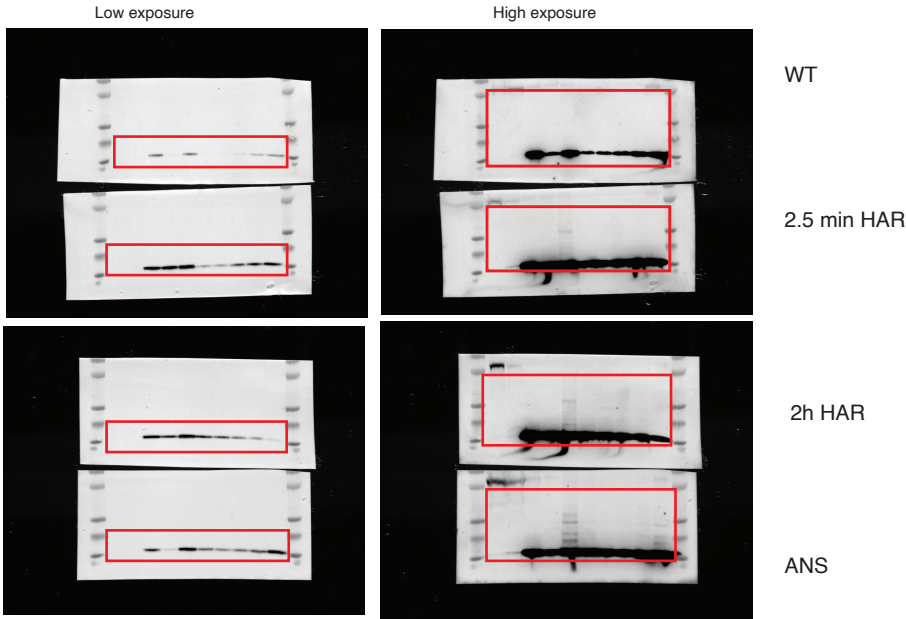

Supplement: Supplementary file 11 — Uncropped western blots. [file 41594_2025_1616_MOESM11_ESM.pdf]

Source Data Extended Data Fig. 1a

anti-HA

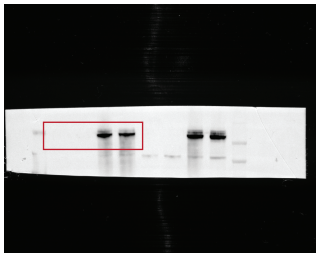

Ponceau (total protein)

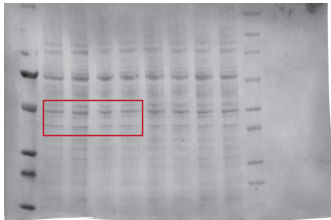

Supplement: Supplementary file 13 — Uncropped western blots. [file 41594_2025_1616_MOESM13_ESM.pdf]

Source Data Extended Data Fig. 2g

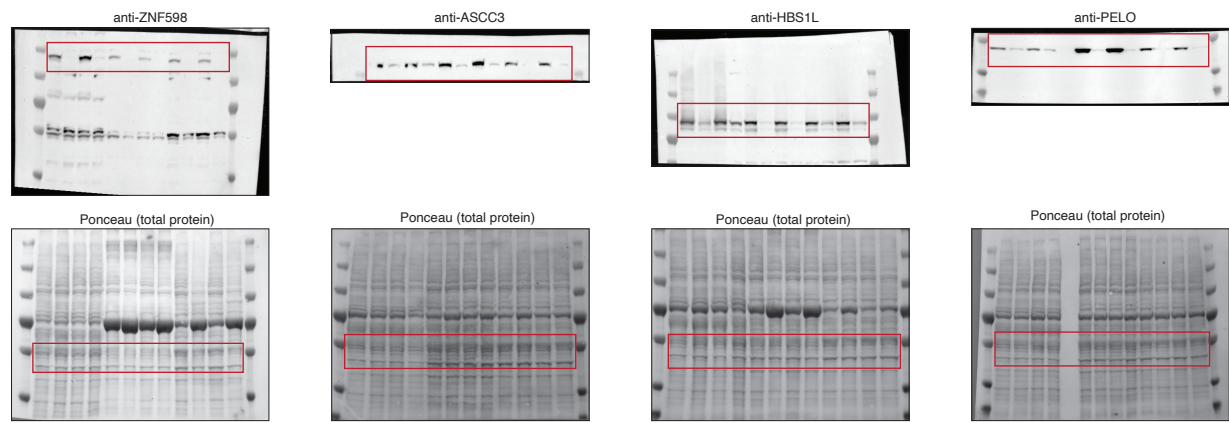

Supplement: Supplementary file 15 — Uncropped western blots. [file 41594_2025_1616_MOESM15_ESM.pdf]

anti-ZNF598

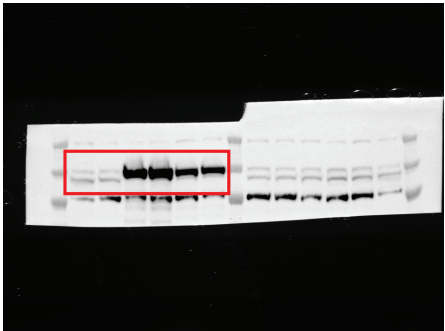

anti-HA

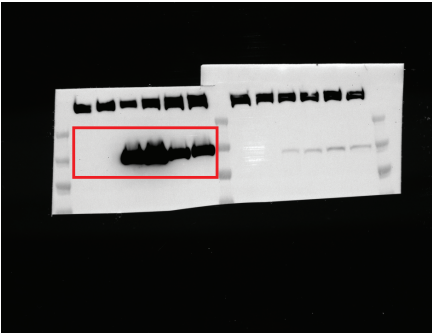

Ponceau (total protein)

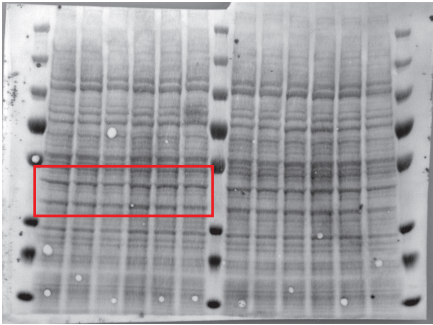

Ponceau (total protein)

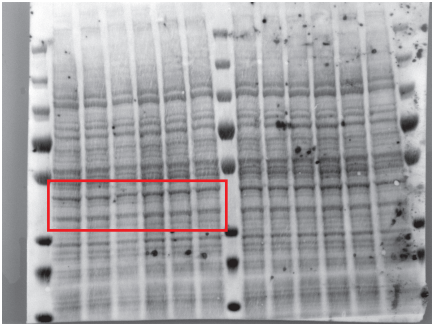

Supplement: Supplementary file 20 — Uncropped western blots. [file 41594_2025_1616_MOESM20_ESM.pdf]
