# Supplementary material for: Comparative CRISPRi screens reveal a human stem cell dependence on mRNA translation-coupled quality control
Source: Nat Struct Mol Biol. 2025 Jul 11;32(10):1932–46. doi: 10.1038/s41594-025-01616-3 (PMC12527931; doi:10.1038/s41594-025-01616-3)
Supplement: Supplementary file 23 — Uncropped western blots. [file 41594_2025_1616_MOESM23_ESM.pdf]

Source Data Extended Data Fig. 8f

anti-eS10

Low exposure | High exposure

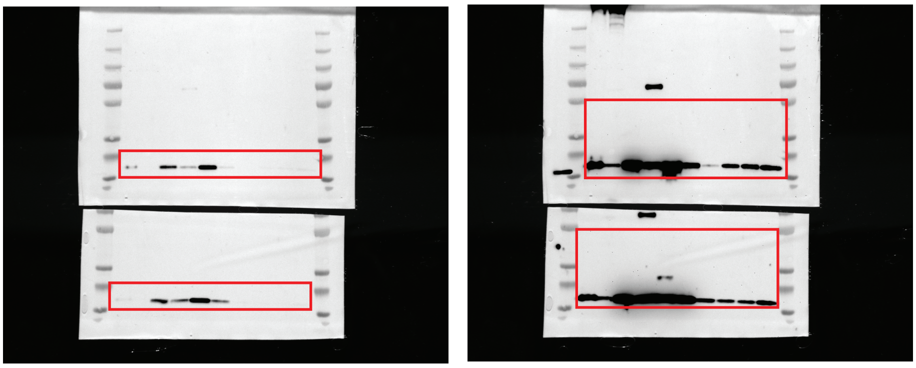

WT

2.5 min HAR

anti-uS10

Low exposure | High exposure

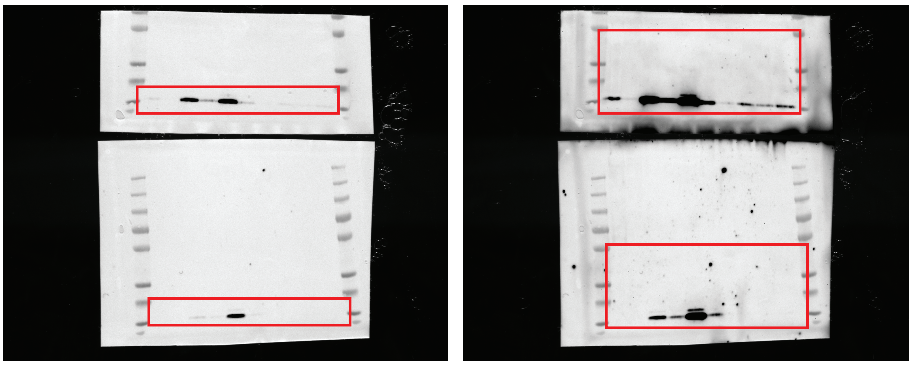

WT

2.5 min HAR

Source Data Extended Data Fig. 8g

anti-uS3

Low exposure | High exposure

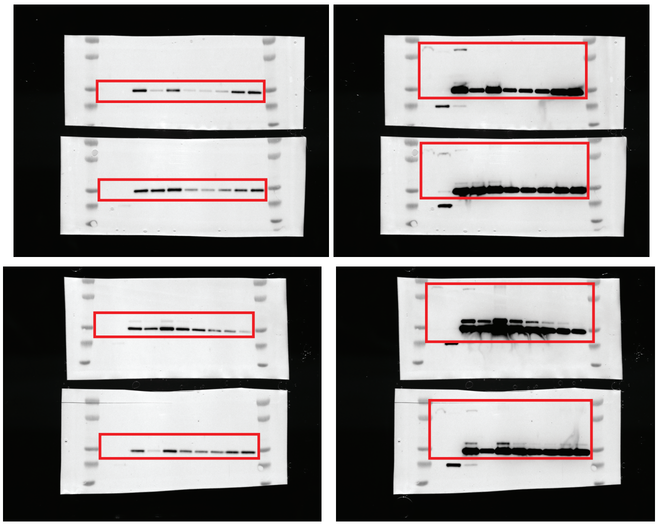

WT

2.5 min HAR

2h HAR

ANS

anti-uS5

Low exposure | High exposure

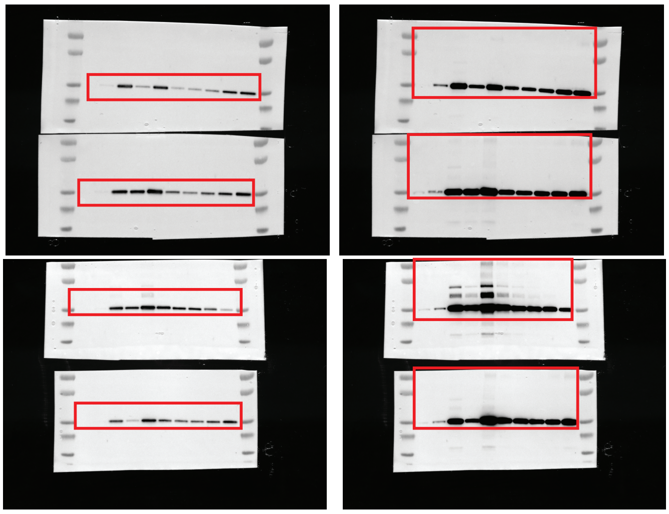

WT

2.5 min HAR

2h HAR

ANS
